# Conversion of MgO nanocrystal surfaces into ceramic interfaces: Exsolution of BaO as photoluminescent interface probes

**DOI:** 10.1111/jace.18833

**Published:** 2022-10-26

**Authors:** Thomas Schwab, Hasan Razouq, Korbinian Aicher, Gregor A. Zickler, Oliver Diwald

**Affiliations:** ^1^ Department of Chemistry and Physics of Materials Paris‐Lodron Universität Salzburg Salzburg Austria

**Keywords:** BaO, cathodoluminescence, chemical vapor synthesis, nanocrystalline ceramics, photoluminescence, segregation, surface excitons

## Abstract

Ion exsolution can be instrumental to engineer intergranular regions inside ceramic microstructures. BaO admixtures that were trapped inside nanometer‐sized MgO grains during gas phase synthesis undergo annealing‐induced exsolution to generate photoluminescent surface and interface structures. During their segregation from the bulk into the grain interfaces, the BaO admixtures impact grain coarsening and powder densification, effects that were compared for the first time using an integrated characterization approach. For the characterization of the different stages the materials adopt between powder synthesis and compact annealing, spectroscopy measurements (UV–Vis diffuse reflectance, cathodo‐ and photoluminescence [PL]) were complemented by an in‐depth structure characterization (density measurements, X‐ray diffraction [XRD], and electron microscopy). Depending on the Ba^2+^ concentration, isolated impurity ions either become part of low‐coordinated surface structures of the MgO grains where they give rise to a characteristic bright PL emission profile around *λ* = 500 nm, or they aggregate to form nanocrystalline BaO segregates at the inner pore surfaces to produce an emission feature centered at *λ* = 460 nm. Both types of PL emission sites exhibit O_2_ gas adsorption‐dependent PL emission properties that are reversible with respect to its pressure. The here‐reported distribution of BaO segregates between the intergranular region and the free pore surfaces inside the MgO‐based compacts underlines that solid‐based exsolution strategies are well suited to stabilize nanometer‐sized segregates of metal oxides that otherwise would coalesce and grow in size beyond the nanoscale.

## Graphical abstract

States in the process starting with metal oxide nanoparticles in nonequilibrium configurations and ending with functional ceramics that exhibit engineered intergranular regions.

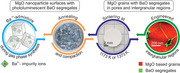

## 1 INTRODUCTION

Advances in synthesis and processing of metal oxide nanocomposites,^1^ in manufacturing of powders and ceramics, and—last but not least—in materials characterization have paved the way to engineer functional interfaces inside polycrystalline materials.^2^, ^3^, ^4^, ^5^, ^6^, ^7^ This is true for a variety of approaches that aim at tuning both the grain boundary region and the free surfaces that define the residual pores. Segregation engineering and the exsolution of admixtures, which can be introduced by the synthesis of nanomaterials as nonequilibrium solids, have attracted increasing attention in this respect.^8^, ^9^, ^10^ Understanding of the structural transformation grains and their interfaces can undergo during ceramics processing and—in particular—during heat treatment are key to gain control over microstructure evolution.^11^

Dispersed barium oxide (BaO) has attracted a great interest due to a variety of applications, above all in heterogeneous catalysis or in electron tube industry.^12^ Potential and use of BaO admixtures in ceramics are manifold. Examples are donor‐doped BaTiO_3_ thermistor ceramics with positive temperature coefficients of resistivity,^13^, ^14^ where the defect chemistry of these interfaces is determined by the local BaO enrichment.^13^, ^14^ Relevant to the field of catalyst and photocatalyst design, Ba^2+^ admixtures to free surfaces and grain boundary interfaces of nanometer‐sized TiO_2_ grains directly impact their coarsening.^15^ As a third example, BaO surface segregates stabilized in Ba^2+^ containing ZrO_2_ or MgO particle powders exhibit great potential as inorganic phosphors, which—depending on the materials processing and excitation profile—produce light emission in the visible light range that can be tuned.^16^, ^17^

Traces of highly dispersed BaO can be employed in ceramic matrices as optical probes. Their absorption and emission properties in the UV–Vis range—via photoexcitation of interface elements—can be measured in the diffuse reflectance (DR) mode or photoluminescence (PL) emission (radiative deactivation of excitons at surfaces and interfaces) mode, respectively. In principle, such nanocrystalline probes could enable one to track interfacial changes like segregation, formation of new interfaces or clustering. Corresponding understanding about structural transformations and associated property changes, in turn, is important to control sintering processes and microstructural developments that lead to ceramics and new catalysts.^18^, ^19^

**FIGURE 1:**
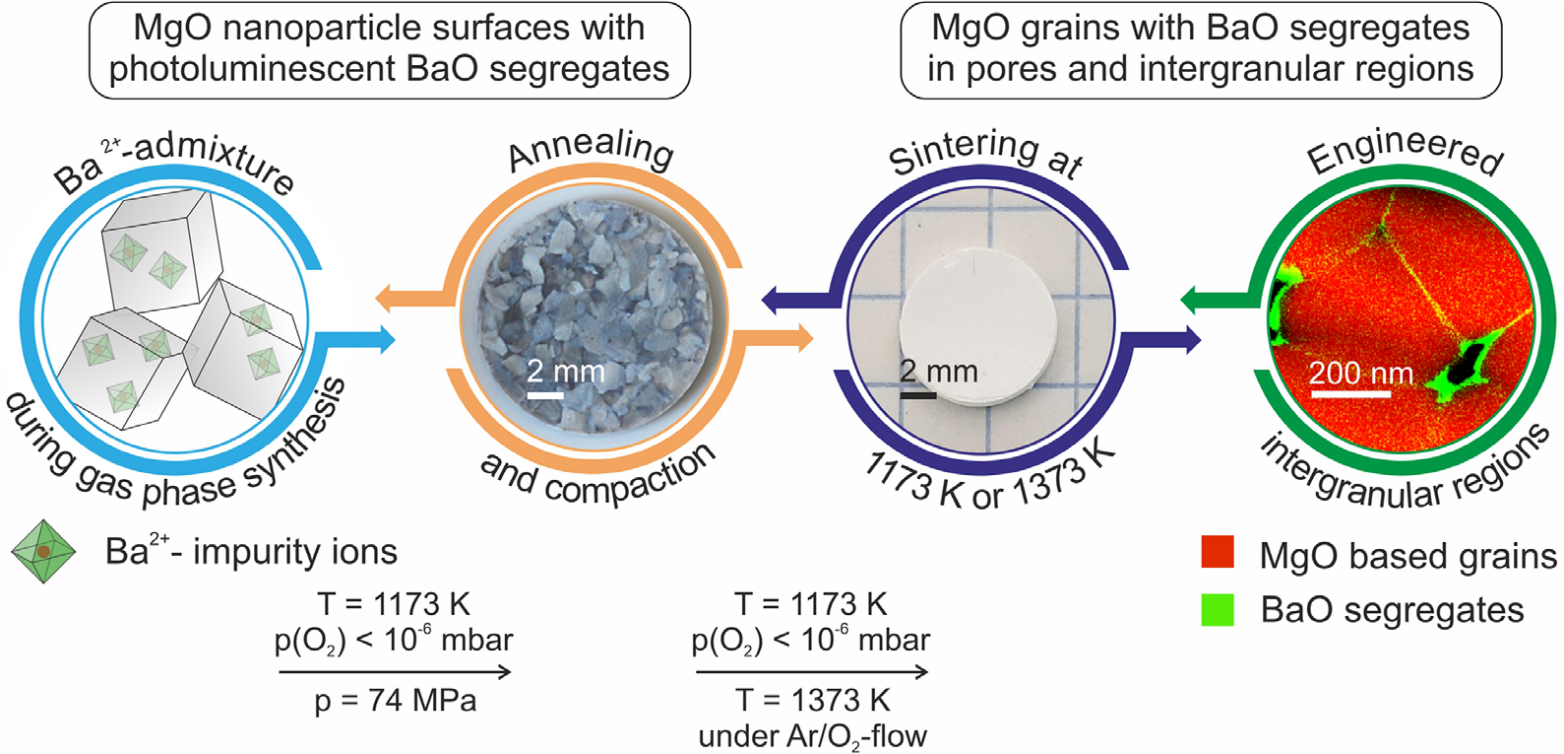
Approach to address the properties of materials at different stages of ceramic processing. Vapor phase grown nanoparticle powders with statistically distributed Ba^2+^ dopants inside the MgO host matrix are converted into ceramics with functionalized intergranular regions. The processing protocol includes vacuum‐based surface purification of the particle powders, powder compaction, and subsequent sintering.

From a chemical perspective, highly dispersed BaO exhibits a remarkable surface basicity. When accessible to gaseous or condensed water from the atmosphere, such surface decorations can induce substantial reactivity changes at room temperature, which were found to directly affect particle size distribution (PSD) and morphology.^20^ Based on a previous analysis of the optical properties of Ba‐doped MgO nanoparticle powders, we now use these impurities as optical probes to track the transformation of free nanoparticle surfaces into interfaces that emerge upon powder compaction and sintering in nano‐ or microcrystalline ceramics. We also explored whether the PL properties specific to highly dispersed BaO, either as Ba–O moieties embedded in an MgO‐based interface structure or as BaO clusters, can be retained after their incorporation into the intergranular region of consolidated and sintered MgO ceramics. The experimental structure of this study is outlined in Figure 1.

We report the structural and optical properties of Ba‐doped MgO nanoparticles prior to and after compaction by uniaxial pressing and analyzed the dependence of PL properties on Ba‐loading for the sintered pellets. Our systematic study does also include a comparison of spectroscopic results obtained either under high‐vacuum conditions or in the presence of molecular oxygen. The latter atmosphere is known to quench surface exciton states like those observed for alkaline‐earth oxides^21^ and provides important information about the accessibility of photoluminescent sites to molecules from the gas phase.

## 2 EXPERIMENTAL PROCEDURES

### 2.1 Ba*_x_* Mg_1−_ *_x_* O nanoparticle powder synthesis

**FIGURE 2:**
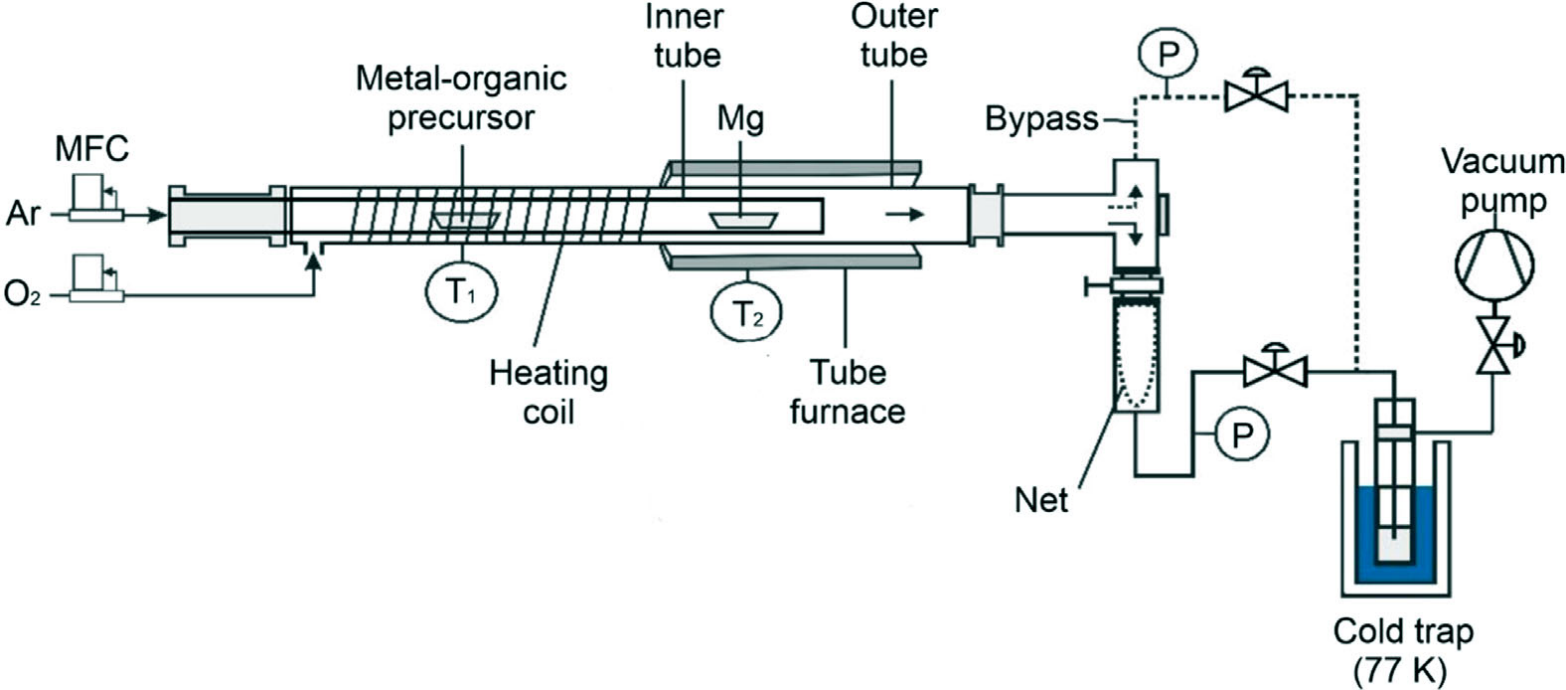
Schematic illustration of the modified tubular hot‐wall reactor setup for the gas phase synthesis of Ba*_x_* Mg_1−_ *_x_* O nanoparticles. *Source*: Adapted from Ref. [22]

We produced^18^ Ba*_x_* Mg_1−_ *_x_* O nanocrystals via a hybrid metal–organic chemical vapor synthesis approach that is described in detail elsewhere.^22^, ^23^, ^24^ We used a reactor consisting of two concentrically arranged quartz glass tubes (Figure 2).

Two precursor containers were placed at different positions inside an inner tube. The first ship contains the metal–organic precursor (barium (2,2,6,6,‐tetramethyl‐3,5‐heptanedionate) Ba(TMHD)_2_). At this position, the tube is surrounded by a heating coil, which is heated to *T*_1_ = 493, 503, or 513 K to sublimate the metal–organic precursor (first heating zone) and to adjust different Ba‐concentrations inside the sample. The second ship contains metallic magnesium turnings (Mg, 99.98%, Alfa Aesar). At this position, the tubes are surrounded by a tube furnace that is heated to *T*_2_ = 913 K (second heating zone) to guarantee the sublimation of the metallic Mg to the gas phase. An argon gas flow (Ar 5.0, volumetric flow rate *Q*_Ar_ = 1200 sccm) flows through the inner tube to transport the metal–organic vapor from the first heating zone to the second heating zone. The metal–organic vapor becomes mixed and decomposed within the magnesium vapor. The vapor mixture is then transported by the argon gas flow to the end of the inner glass tube, where the vapor mixture gets in contact with molecular oxygen (O_2_ 5.0, *Q*_O2_ = 1200 sccm) from the outer tube. Barium precursor decomposition inside the combustion flame of Mg vapor leads to subsequent nanoparticle formation. Continuous pumping to *p* = 70 ± 1 mbar keeps the residence time of resulting nanoparticles within the flame short. This prevents undesired particle coarsening and coalescence. Nanoparticle agglomerates are transported through the gas flow to be collected via a stainless steel net. The total pressure, gas flows, and furnace temperature are kept constant during the entire period of particle production.

### 2.2 Annealing treatment

After production, the Ba*_x_* Mg_1−_ *_x_* O nanoparticle powders were transferred into quartz glass cells that allow thermal powder activation in defined gas atmospheres. The as‐synthesized powder was subjected to stepwise heating to the final temperature (873, or 1173 K) with a heating rate of 5 K per min in high‐vacuum conditions *p* < 10^−5^ mbar. This heating step eliminates the adsorbed water and other volatile substances. Subsequent exposure to molecular oxygen and evacuation at these temperatures was performed to eliminate carbon remnants from the metal–organic precursor by converting them into volatile CO and CO_2_.

### 2.3 Powder compaction and annealing

Powder compaction was performed via cold uniaxial pressing resulting in a disk‐shaped specimen. A defined mass of powder (*m* = 150 ± 10 mg) was transferred into the cavity (*d* = 13 mm) of a compaction tool (FTIR Pellet Dies, Specac) and hydraulically consolidated by applying the pressure of 74 MPa for 1 min to obtain green compacts. We performed thermal treatment using a muffle furnace (Nabertherm LT 5/12). This furnace provides the possibility to control the sintering atmosphere by introducing a continuous gas flow. The green compact was placed in the muffle furnace inside an Al_2_O_3_ sample container and heated up to 1173 K. This step was performed under continuous oxygen flow and with a heating rate of 10 K/min. The oxygen flow at atmospheric pressure is provided during the heating to purify the sample from the adsorbed species, which arise from processing in the ambient atmosphere. After reaching 1173 K and dwelling for 10 min, the oxygen flow was replaced by continuous argon flow, and the sample was heated at 10 K/min to the final temperature of 1373 K. This temperature was kept constant for 2.5 h before cooling down to room temperature.

### 2.4 Structure characterization

X‐ray diffraction (XRD): XRD data was collected at room temperature in coupled theta–theta mode on a Bruker AXS D8 ADVANCE diffractometer. Powder samples and ceramic specimens, dry‐ground in a porcelain mortar to ensure sufficient diffraction intensity, were prepared on a single‐crystal silicon zero‐background sample holder. Data acquisition was done using Cu *K*_α_ 1,2 radiation (*λ* = 154 pm) between 5° and 80.5° 2*θ* with a step size of 0.02° and opened divergence and anti‐scatter slits at 0.3° and 4°, respectively. A primary and secondary side 2.5° Soller slit was used to minimize axial divergence, and a detector window opening angle of 2.93° was chosen. Data handling and qualitative phase analysis were performed with the Bruker software DIFFRAC.EVA V2.1. Crystallite domain sizes were determined from powder diffraction data using the Scherrer equation.

Electron microscope images were acquired using scanning electron microscopy (SEM) and transmission electron microscopy (TEM). The SEM (Zeiss Fe‐Ultra Plus 55) was equipped with a field emission gun, Gemini lenses, and an Oxford Instruments EDX‐silicon drift detector (50 mm^2^, energy resolution <127 eV @ Mn *K*_α_). The TEM (JEOL JEM‐F200 TEM) was operating at 200 kV and equipped with a cold field‐emission electron source, a large windowless JEOL Centurio EDX detector (100 mm^2^, 0.97 srad, energy resolution <133 eV), and a TVIPS F216 2k by 2k CMOS TEM camera. A TEM image analysis was performed with the EM Measure software from TVIPS. The ceramic samples were prepared for TEM investigation by gradually grinding and further thinning by an ion polishing system. Grinding was performed with the help of a precision polishing system (Allied MultiPrep TM). The ion polishing system employed was a precision ion polishing system II—Model 695 (GATAN).

### 2.5 Spectroscopy

For the spectroscopy measurements, the samples were placed in special quartz glass cells, where measurements can be performed without breaking the vacuum and under defined gas atmospheres. UV–vis DR spectra were recorded with a PerkinElmer LAMBDA 750 with an integrating sphere detector to determine the absorption spectra of the samples. The measurements were carried out under an oxygen atmosphere *p*(O_2_) = 100 mbar to quench surface excitons–related luminescence from particle surfaces. The recorded DR spectra were transformed into the Kubelka–Munk function. PL spectra were recorded using an Edinburgh Instruments FLS 980 spectrometer equipped with a double grating monochromator system on both the excitation and the emission site. As a light source, a 450 W ozone‐free continuous‐wave xenon arc lamp was used. The detector was an R928P from Hamamatsu working at 253 K for optimal dark count reduction. For the luminescence measurements, the samples were measured in a high vacuum, *p* < 10^−5^ mbar, to avoid quenching effects. Cathodoluminescence (CL) measurements were performed inside the previously mentioned SEM with the Mono CL4 system from GATAN. The powder samples were attached to the sample holder using carbon tape. The measurements were performed using the electron beam of the SEM with an acceleration voltage of 20 kV. Evaluation of the data was performed with the help of the software DigitalMicrograph from GATAN.

## 3 RESULTS AND DISCUSSION

### 3.1 Ba exsolution in vacuum annealed nanoparticle powders

**FIGURE 3:**
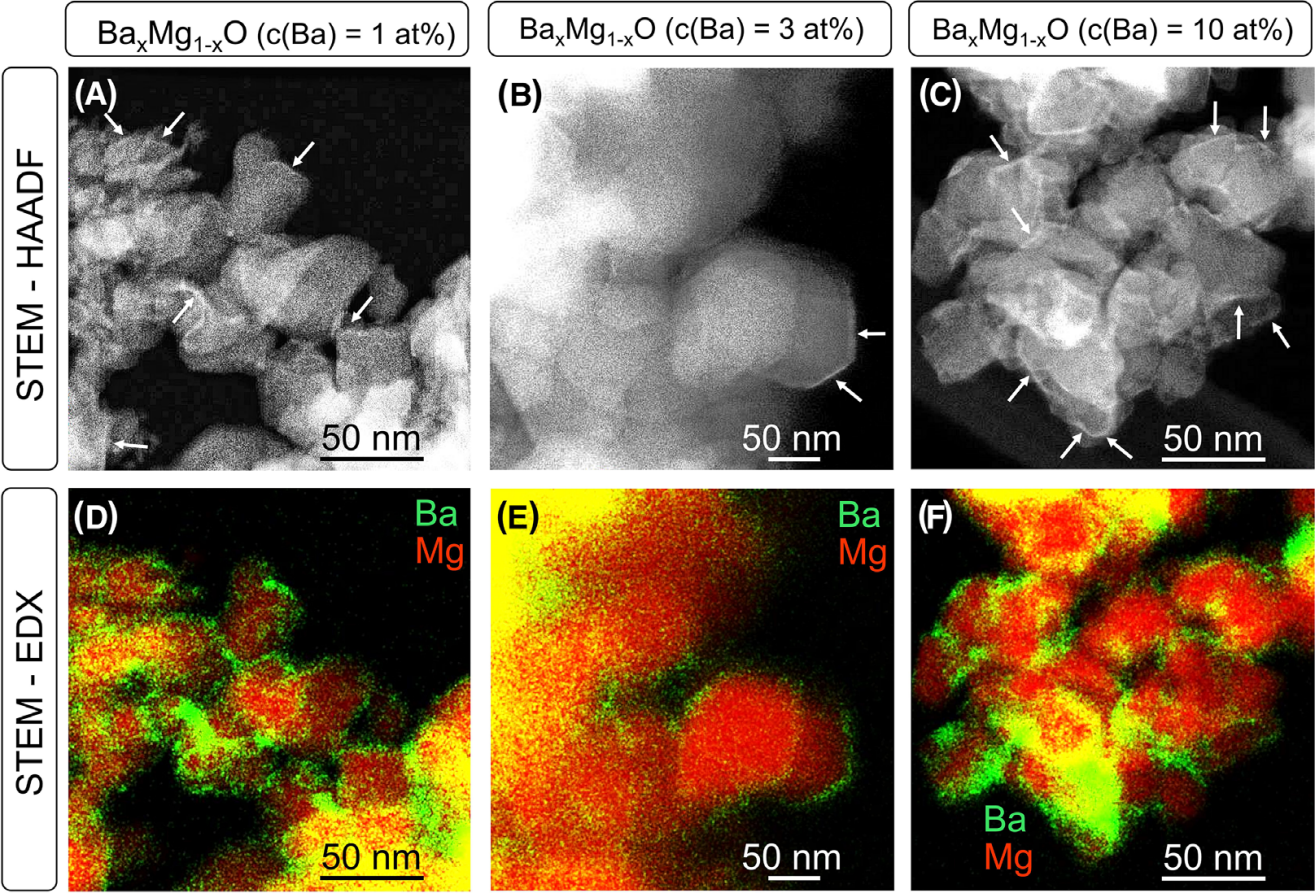
Annealing‐induced Ba‐exsolution generates BaO surface layers and coarsened BaO segregates (see also Figure S2). The annealing temperature was 1173 K for Ba*_x_* Mg_1−_ *_x_* O samples with Ba‐concentrations of 1 at% (left column A, D), 3 at% (middle column B, E), and 10 at% (right column C, F). The STEM–HAADF images (top row, A–C) reveal the compositional contrast between the brighter Ba‐rich segregates (indicated by arrows) and the MgO‐based host particles. Corresponding STEM–EDX intensity maps (bottom row, D–F) confirm Ba‐accumulation at nanoparticle surfaces and Ba‐depletion in the bulk region of the MgO nanocrystals (red: Mg, green: Ba).

Ba*_x_* Mg_1−_ *_x_* O nanoparticles, where the admixed Ba^2+^ ions during synthesis are statistically distributed over the MgO host grains of the as‐synthesized powders, were obtained by chemical vapor synthesis that is based on a recently developed reactor design.^22^, ^23^, ^25^ Electron microscopy (SEM/EDX and STEM/EDX) was used for the analysis of the chemical composition. Ba^2+^ concentrations of 1, 3, and 10 at% were measured for the annealed nanoparticle powders (Table S1 and Figure S1). In addition, annealing‐induced Ba segregation was confirmed by STEM–HAADF measurements and EDX mappings (Figure 3).

In general, the brighter regions in the STEM–HAADF images arise from thickness contrast. However, the contrast of certain regions with homogeneous thickness in Figure 3, which are indicated by arrows, is determined by the higher *z*‐contrast of Ba‐rich segregates. This is consistent with STEM–EDX intensity maps (Figure 3D–F). (The formation of Ba‐rich surface features results from annealing at *T* ≥ 873 K [see also Figure S2].) Thin BaO surface layers as well as thicker and crystalline BaO‐rich surface segregates, the relative abundance of which increases with BaO concentration and annealing temperature, are the two major types of BaO‐related surface features observed.

### 3.2 Evolution of particle morphology and size distribution in powders with annealing

**FIGURE 4:**
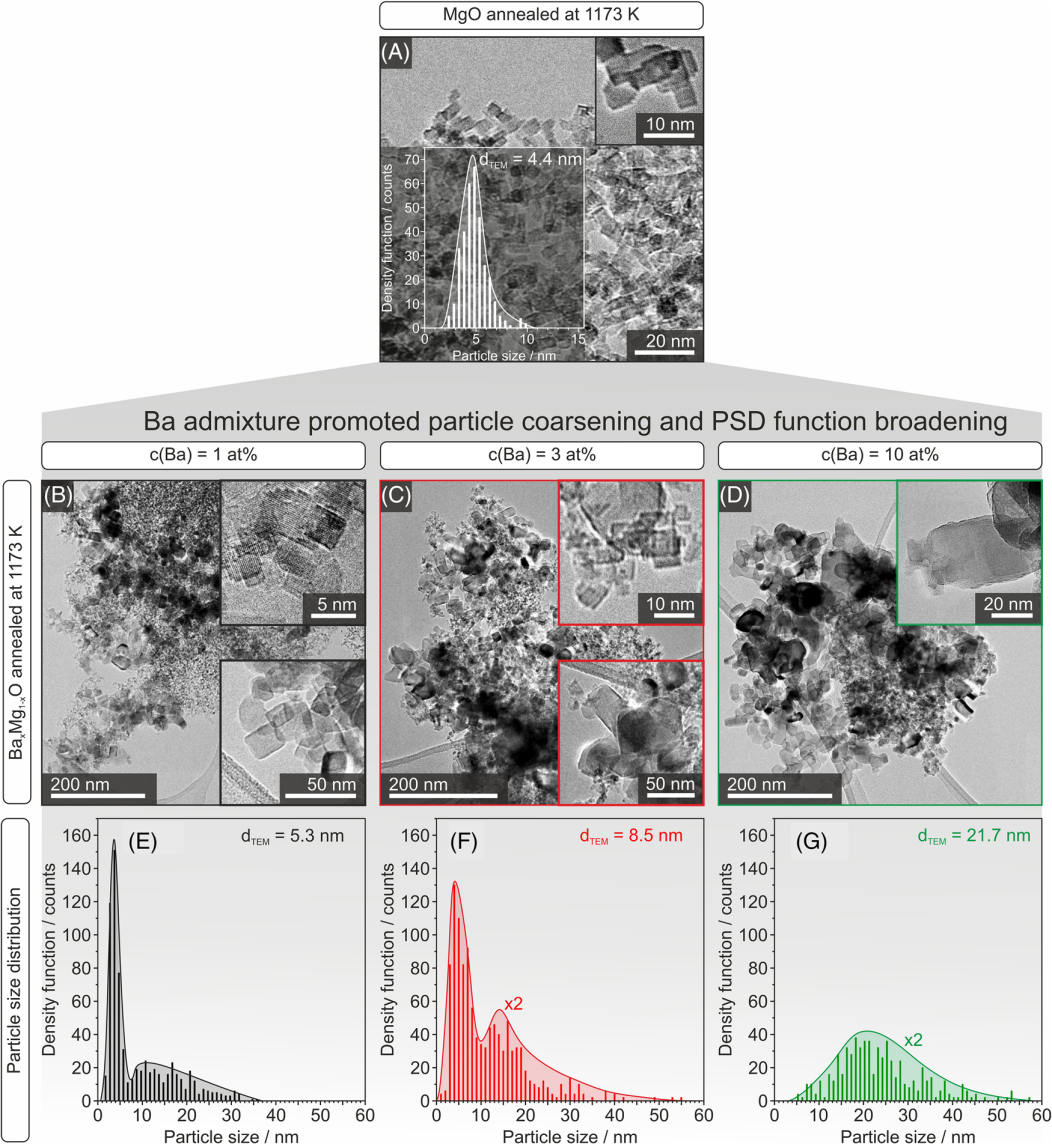
Transmission electron micrographs of MgO nanocrystals (A, top) and Ba*_x_* Mg_1−_ *_x_* O nanocrystals with the corresponding particle size distribution (PSD) plots (second and third row, B–G) with Ba‐concentrations of 1 at% (left column B, E), 3 at% (middle column C, F), and 10 at% (right column D, G). Powder annealing to 1173 K of particle ensembles with Ba‐admixtures promotes coarsening and disproportionation effects related to the PSD. The insets in B, C, and D provide information about the typical particle morphologies.

Annealed powder samples of MgO and Ba*_x_* Mg_1−_ *_x_* O (*c*(Ba) = 1, 3, 10 at%) nanoparticles were analyzed with TEM. The representative compilation of data in Figure 4 underlines the impact of Ba^2+^ admixture on particle size and morphology evolution.

After annealing to 1173 K, MgO nanocubes retain their narrow and monomodal size distribution with a median value of 4.4 nm (Figure 4A top). In contrast, Ba‐admixtures‐affected annealing‐induced changes in grain morphology lead to a shift of the grain size distribution toward larger values. For Ba‐concentrations of 1 at% (Figure 4B) and 3 at% (Figure 4C), we were still able to observe cubic particles (top right insets in Figure 4B,C) with sizes below 10 nm. In addition, we also identified larger particles (*d* > 10 nm) with truncated edges and generally less‐defined morphologies (bottom right insets in Figure 4B,C). The fraction of larger particles has become apparent in the corresponding PSD plots (Figure 4E,F), which reach sizes up to 50 nm and which show broad and multimodal PSD with median values of 5.3 and 8.5 nm for samples with Ba‐concentrations of 1 and 3 at%, respectively. For samples with 10 at% Ba‐admixture, there exists no fraction of particles with sizes below 10 nm. Exclusively larger particles characterized by a broad size distribution with a median value of 22 nm and with grains of a less‐defined morphology (inset in Figure 4D) were found. (These observations are different from those obtained on corresponding samples after annealing to temperatures not higher than 873 K. The monomodal size distributions with median values below 10 nm are retained for annealing at 873 K (Figure S3).)

Changes in particle morphology and size distribution originate from the enhanced ion diffusion inside the Ba^2+^‐doped MgO lattice in combination with Ba^2+^‐segregation into the nanoparticle surfaces (Figures 3 and S2). The enhanced ion diffusion of Ba^2+^ ions is mainly driven by the size mismatch between octahedrally coordinated Ba^2+^ (0.135 nm) and Mg^2+^ (0.072 nm).^20^, ^24^, ^26^, ^27^, ^28^ The onset of Ba segregation can be observed for annealing at 873 K (Figure S3) where slight but significant particle coarsening gives rise to a final median size that still remains in the range below 10 nm. Annealing in the temperature range between 873 and 1173 K, however, enforces ion diffusion and strongly promotes coarsening and disproportionation in the PSD.

**FIGURE 5:**
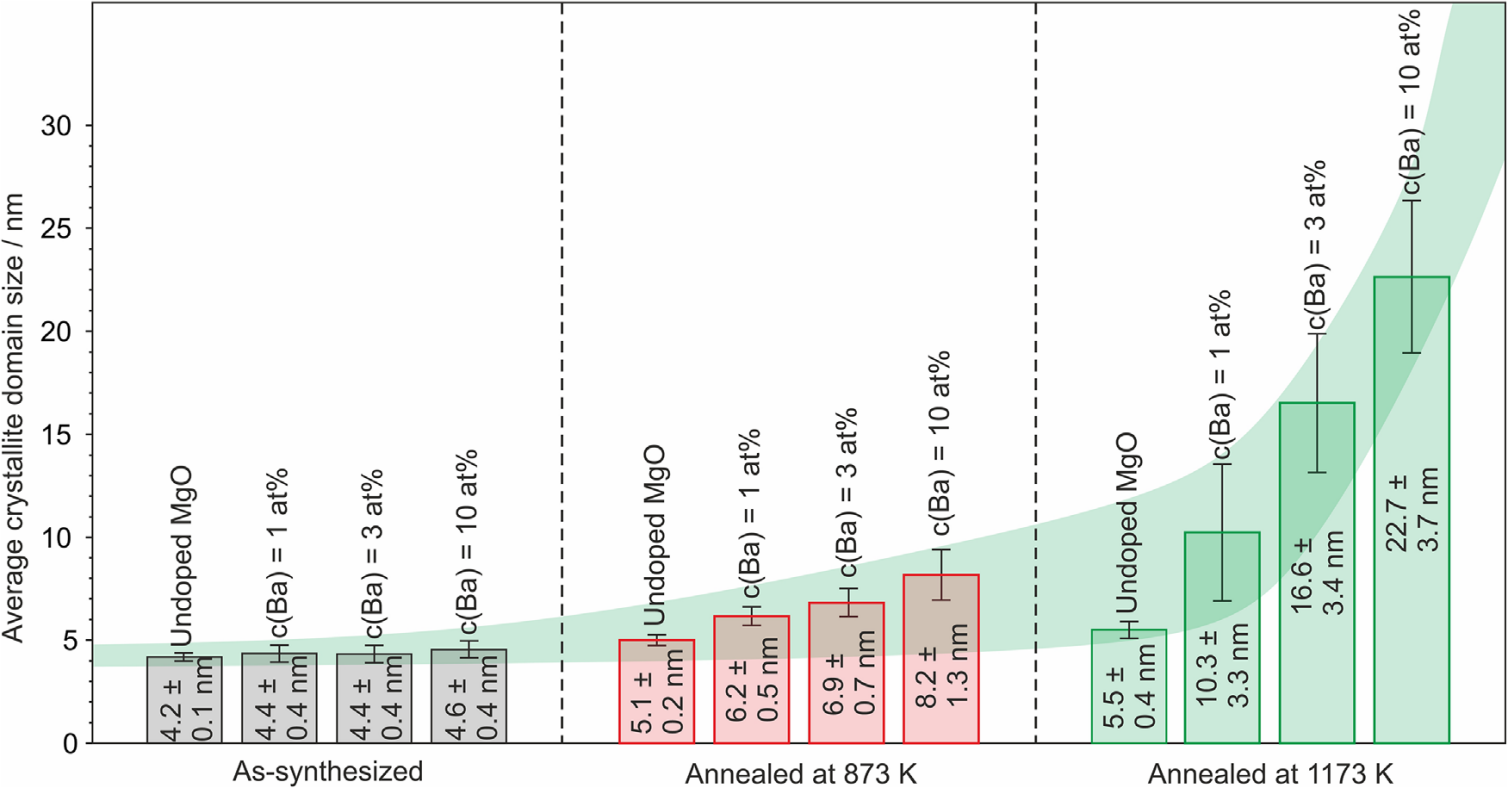
Average crystallite domain size values determined for MgO nanoparticle ensemble with different Ba^2+^ concentrations. The values were obtained via the analysis of the widths of the MgO(2 0 0) diffraction features using the Scherrer equation.

Additional contributions to the particle growth of the incorporated and segregating Ba^2+^ ions involve the enhanced interface reactivity of related segregates and an associated enforcement of the contact among the individual MgO‐based grains. This, in turn, promotes mass transport. A detailed mechanism cannot be established on the basis of the available data. We complemented the insights from TEM analysis with XRD measurements and tracked the influence of Ba^2+^ admixture on the diffraction features that are specific to the MgO‐based host grains (Figure S5). We did not observe any shifts of the MgO‐specific diffraction features that would indicate strain effects as one would expect from the inclusion of Ba–O moieties inside the MgO host lattice.^29^ The diffraction features’ linewidth, however, decreased with Ba^2+^ concentration at a given annealing temperature and with annealing temperature. Underlying crystallite domain size changes confirm the coarsening effects that are consistent with the PSD plots discussed along Figure 4. Using the Scherrer equation, we determined that the grain size growth and related trends are plotted in Figure 5.

We observed low‐intensity XRD lines that are attributed to Ba(OH)_2_·H_2_O, BaO_2_·(H_2_O_2_), and BaCO_3_ for samples with Ba^2+^ concentrations of 3 at% and larger after powder annealing and the subsequent and unavoidable exposure to air (Figure S4b,c). Hydroxides and carbonates must form instantaneously upon sample exposure to air.^20^ Although in case of the XRD measurements sample transfer of the XRD diffractometer exposure to air is unavoidable, the microscopy and spectroscopy studies were exclusively performed under high‐vacuum conditions and on materials that were previously annealed to temperatures above the decomposition temperature of hydroxides and carbonates, that is, to 1173 K. Thus, the spectroscopic data reported below must be related to grain ensembles with dehydroxylated grain surfaces that are exclusively composed of Mg^2+^, Ba^2+^, and O^2−^ ions.

### 3.3 Optical powder properties—UV diffuse reflectance and photoluminescence emission

**FIGURE 6:**
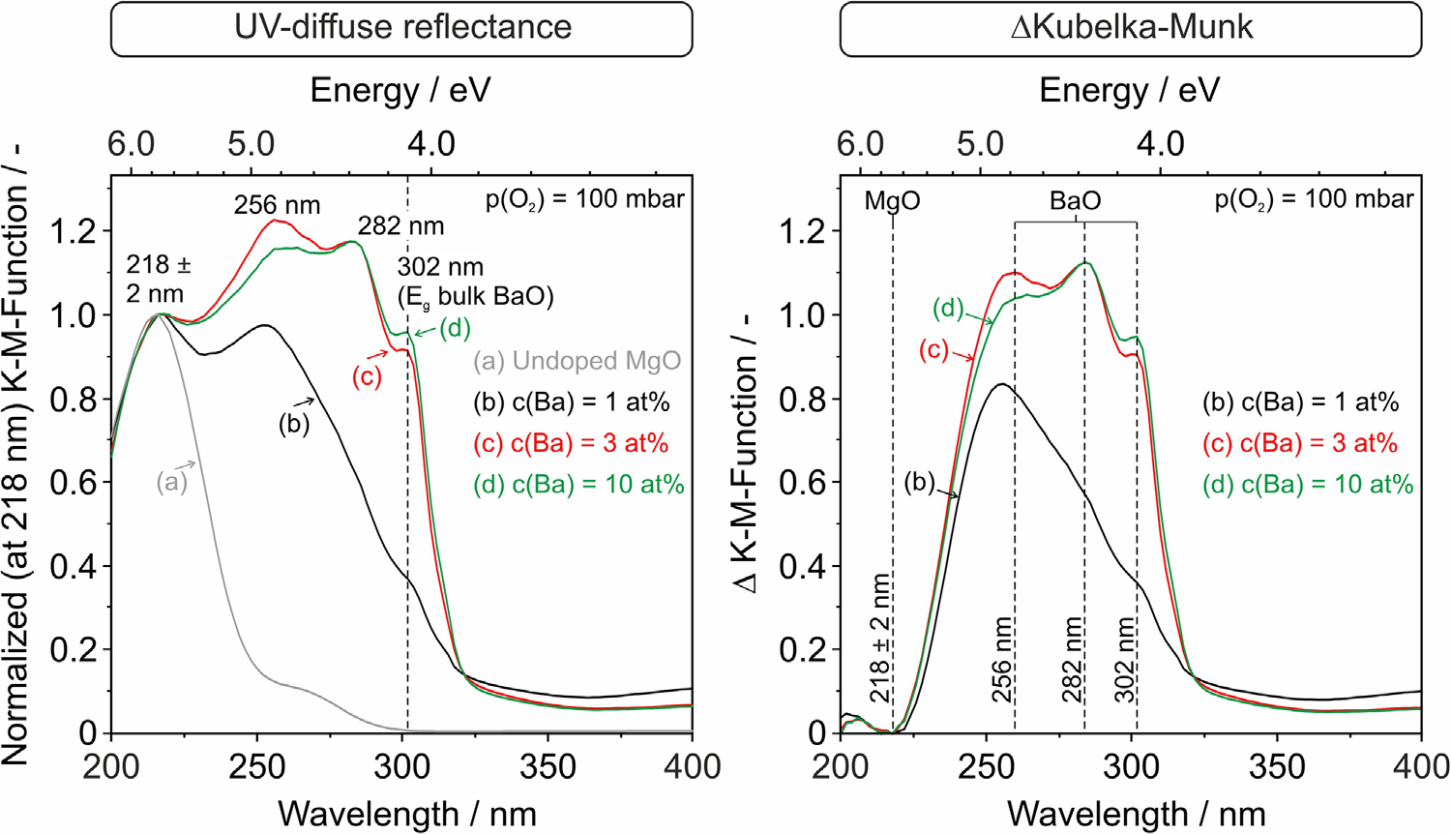
UV diffuse reflectance spectra (left) of MgO nanocube and Ba*_x_* Mg_1−_ *_x_* O nanoparticle powders with Ba‐concentrations of 1 at% (B), 3 at% (C), and 10 at% (D). The spectra were acquired in O_2_ atmosphere (*p*(O_2_) = 100 mbar) and at room temperature after sample annealing to 1173 K. All *K*–*M* functions were normalized to a characteristic MgO absorption feature at 218 ± 2 nm (5.6–5.7 eV).^27^, ^30^ Resulting Kubelka–Munk difference spectra (right panel) reveal optical property changes that arise from segregated Ba^2+^ admixtures.

**FIGURE 7:**
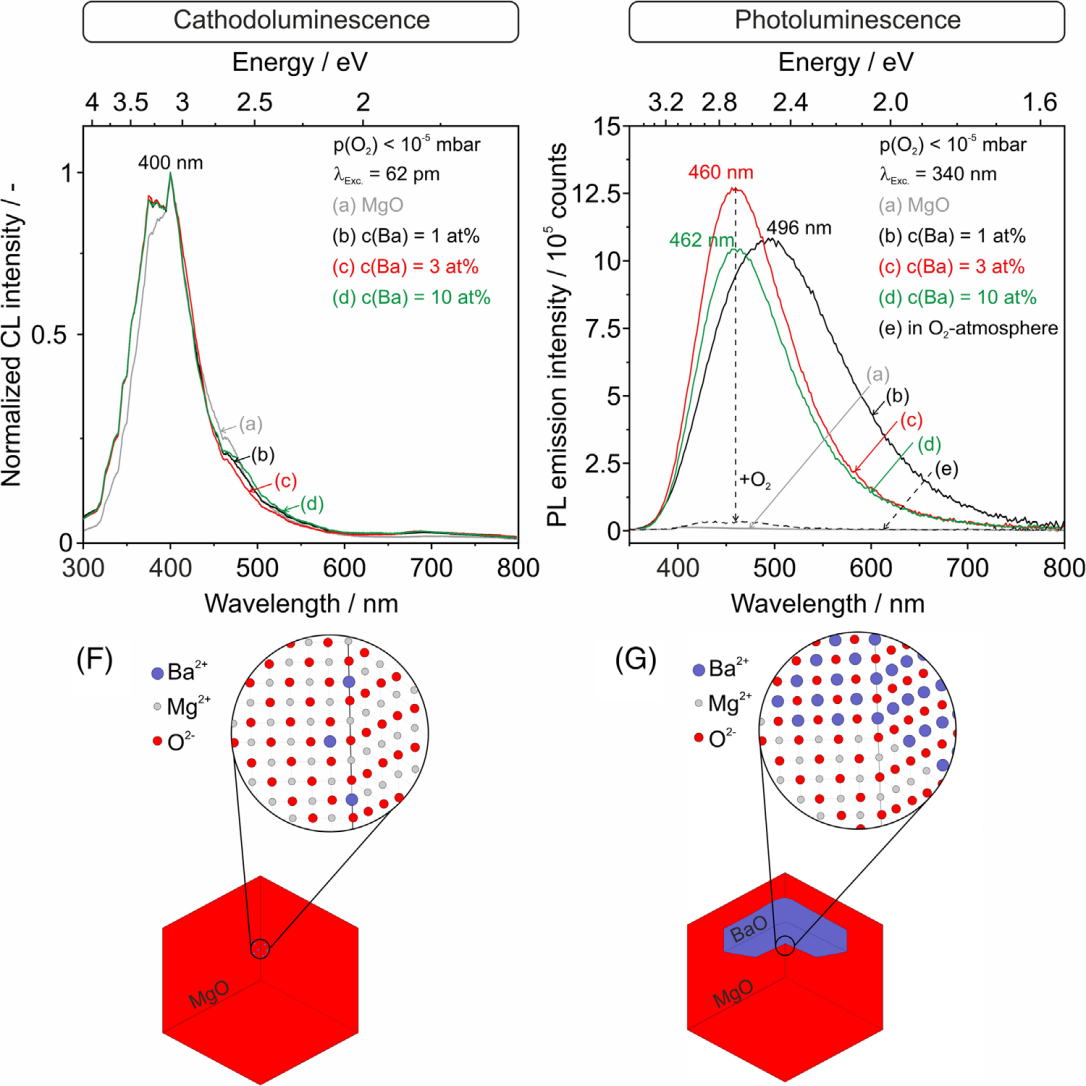
(a–d) Cathodoluminescence (CL, left panel) and (a–e) photoluminescence (PL, right panel) spectra that were acquired at room temperature and in high vacuum (*p* < 10^−5^ mbar). The CL spectra were measured inside the scanning electron microscopy (SEM) chamber and with 20 keV electrons as excitation source. The excitation wavelength for the PL measurements was 340 nm. The schemes in labels (F) and (G) illustrate the two situations where Ba^2+^ ions are isolated and distributed over the surface structure of the MgO‐based grains (F) or where they form nanocrystalline segregates with BaO‐specific optical properties (G).

The significant impact of BaO admixture on the optical powder properties is revealed by UV diffuse reflectance (DR) (Figure 6) and photo‐/CL spectroscopy measurements (Figure 7). For the determination of optical absorption, the UV DR measurements on the nanoparticle powders were acquired in O_2_ atmosphere (*p*(O_2_) = 100 mbar) to avoid artifacts that may arise from PL emission and —after spectra normalization to the MgO‐specific feature at 218 ± 2 nm—to also generate difference spectra (Figure 6, right panel) for the identification of the different Ba‐related absorption features.

Two features in the UV range characterize the optical absorption spectrum of highly dispersed MgO nanocubes (left panel in Figure 6): an absorption maximum at 218 ± 2 nm (5.6–5.7 eV) and a shoulder at 270 nm (4.6 eV). Related bands are attributed to the excitation of four‐ and threefold coordinated O^2−^ located on cube edges and corners, respectively^21^ (Sternig et al. 2015). An admixture of Ba with a concentration of *c*(Ba) = 1 at% (Figure 6B) generates additional absorption features above 220 nm (5.6 eV). Admixture concentration increase toward 10 at% (Figure 6B,C) generates an absorption edge at 4.1 eV (302 nm) that is consistent with the optical bandgap of BaO.^27^ After annealing‐induced Ba exsolution, all these changes originate from the Ba‐enriched surface segregates that remain stabilized at the MgO grain surfaces (Figure 2). The difference spectra in the right panel of Figure 6 clearly reveal the bands with maxima at 256 nm (4.8 eV) and 282 nm (4.4 eV). These arise from highly dispersed BaO clusters as well as mixed Ba–O–Mg surface structures, where the Ba^2+^ ions in the surface region of the MgO‐based grains are well separated from each other. We investigated the radiative decay of the excitonic states that can be probed with CL (Figure 7, left panel) and PL (Figure 7, right panel) spectroscopies.

Irrespective of whether the MgO grains were functionalized with Ba‐admixtures or not, high‐energy excitation with 20 keV electrons (*λ*_exc_ = 8.6 pm) generates very similar emission profiles with a maximum at 400 nm (Figure 7, left panel). This light emission signal is attributed to bulk defects that are present in pure MgO as well as in Ba*_x_* Mg_1−_ *_x_* O grains and, thus, not affected by the Ba‐admixtures. The PL emission spectra, on the contrary, are Ba–O specific, as the excitation wavelength of *λ*_exc_ = 340 nm was used.^12^, ^27^ Related emission features exclusively stem from Ba‐related surface and interface structures and show no resonance with the MgO substrate.

Inside the PL‐spectrometer, the *λ*  = 340 nm (3.65 eV) light generates intense and broad emission bands with maxima in the visible range of light (Figure 7B–D, right panel). Increasing the Ba‐concentration from 1 to 3 or to 10 at% shifts the PL emission band maximum from 500 nm (Figure 7B) to 460 nm (Figure 7C,D). Admission of gaseous oxygen (*p*(O_2_) = 100 mbar) quenches the emission signal, which demonstrates the surface excitonic nature of these processes.

There are two major effects of Ba‐admixture to the grain surface of MgO‐based grains. At low concentrations, that is, *c*(Ba) = 1 at%, the segregated Ba^2+^ ions substitute Mg^2+^ ions and remain isolated at the grain surfaces at or near to low‐coordinated surface sites such as corners and edges. Such surface structures can serve as both excitation and PL emission sites. The abundance of smaller particles observed for a Ba‐concentration of 1 at% (Figure 3A,D) in combination with a higher level of dispersion—as compared to samples with Ba‐concentrations of *c*(Ba) = 3 and 10 at%—is associated with an increased number of such isolated and Ba‐related excitation and emission sites. This increase in the number and heterogeneity of sites is also reflected by the increased width of the PL emission feature in Figure 7B. The dispersion of larger BaO entities related to samples with *c*(Ba) = 3 and 10 at% favors the formation of nanocrystalline BaO clusters that host low‐coordinated excitation and emission sites that are exclusively BaO specific. We therefore attribute the blueshifted and more narrow PL emission signal with a maximum at 460 nm (Figure 7C,D) to nanocrystalline BaO segregates (Figure 7F), whereas the broader emission profile in the visible light range originates from Ba^2+^ ions embedded in low‐coordinated surface elements that are composed of Mg^2+^ and O^2−^ ions (Figure 7G).^27^

### 3.4 Compaction and sintering of green bodies

**FIGURE 8:**
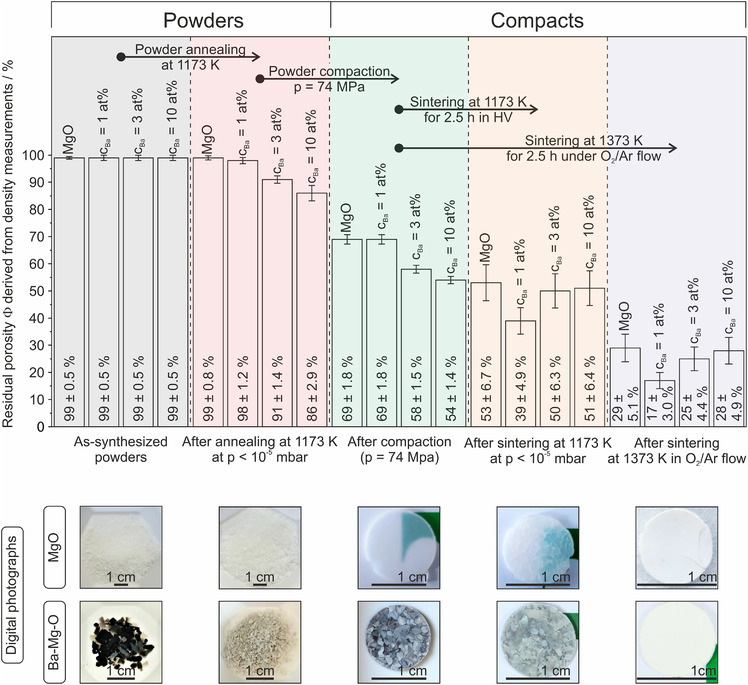
Residual porosities *Φ* derived from density measurements of samples with Ba‐concentrations of 1, 3, and 10 at% and in comparison, to MgO. The values were determined at different stages of ceramic powder processing and the associated digital images of related powders and compacts are at the bottom of the figure. A detailed list of open and closed porosities is provided in Table S2.

In the second part of this study, we addressed the impact of powder compaction and subsequent annealing on grain coarsening, residual porosity, and on the PL emission properties of the BaO segregates inside the resulting microstructures. Density measurements and porosity values derived therefrom (Figure 8) reveal that powder annealing to 1173 K shows an effect only for samples with Ba‐concentrations as high as 10 at%, where Ba‐induced grain coarsening increases the relative densities of the given particle ensembles. Powder compaction—compare the second with the third panel in Figure 8—increases the relative density and, concomitantly, decreases the residual porosity by about−30%. The impact of pressureless sintering can be concluded by comparing the third with the fourth and fifth panels in Figure 8. The compacts residual porosities decrease down to 30% and 50% after vacuum annealing at 1173 K or annealing in alternating O_2_ and Ar atmospheres at 1373 K, respectively.

Complementary XRD measurements indicate that the average crystallite domain sizes remain in the nanocrystalline regime (Figure S6) as long as annealing is performed at 1173 K or below. In principle, one would expect that the mass transport required for grain coarsening and densification depends on the level of consolidation the powder adopts prior to additional heat treatment. Particle contacts and necks represent prominent diffusion paths for interparticulate mass transport during sintering. It is interesting in this context, however, to observe that room‐temperature compaction decreases the residual porosity but does not affect the average particle or grain size after subsequent annealing to 1173 K. A combination of smaller grain size (Figure S6) and grain surface functionalization with Ba at lower Ba coverages may explain the drop in the residual porosity values for nonconsolidated as well as compacted powders of MgO nanocrystals with a Ba‐concentration of 1 at%. In any case, vacuum annealing to 1173 K and above promotes Ba^2+^ exsolution and is effective to eliminate adsorbate species like water, carbonates, or hydroxyls from the grain surfaces when a base pressure of *p* < 10^−5^ mbar is maintained inside the sample cell by continuous pumping.

In this study, all annealing was performed in water‐free gas atmospheres, as an earlier study revealed that the presence of gaseous water strongly affects the MgO grain size, morphology, and microstructure.^31^, ^32^ It promotes mass transport and particle coarsening and stabilizes specific surface modifications such as step edges and shallow protrusions on terraces energetically.^32^ Here, where the grain sizes remain in the nanometer regime, the effect of water‐assisted mass transport and interface modifications could have been decoupled from other contributions to sintering.^17^, ^33^ After annealing to 1373 K in a dry O_2_/Ar atmosphere, the grains undergo strong coarsening, and the small widths of the measured diffraction features render the application of the Scherrer equation impossible. Consequently, the average crystallite domain sizes exceed the 100 nm limit.

Finally, the digital images in the bottom part of Figure 8 provide information about the visual appearance of powders and compacts. The MgO pellet produced by uniaxial powder pressing at 74 MPa is translucent. This translucency is reduced and, ultimately, ceased by subsequent pellet sintering to 1173 and 1373 K, respectively. The dark or brown coloration observed for samples with Ba‐admixtures indicates the presence of coke and carbon residues that originate from the Ba precursor. Annealing in alternating O_2_ and Ar atmospheres to 1373 K perfectly eliminates these impurities and bleaches out the related coloration (last image in the bottom part of Figure 8).

### 3.5 Optical properties of sintered compacts

**FIGURE 9:**
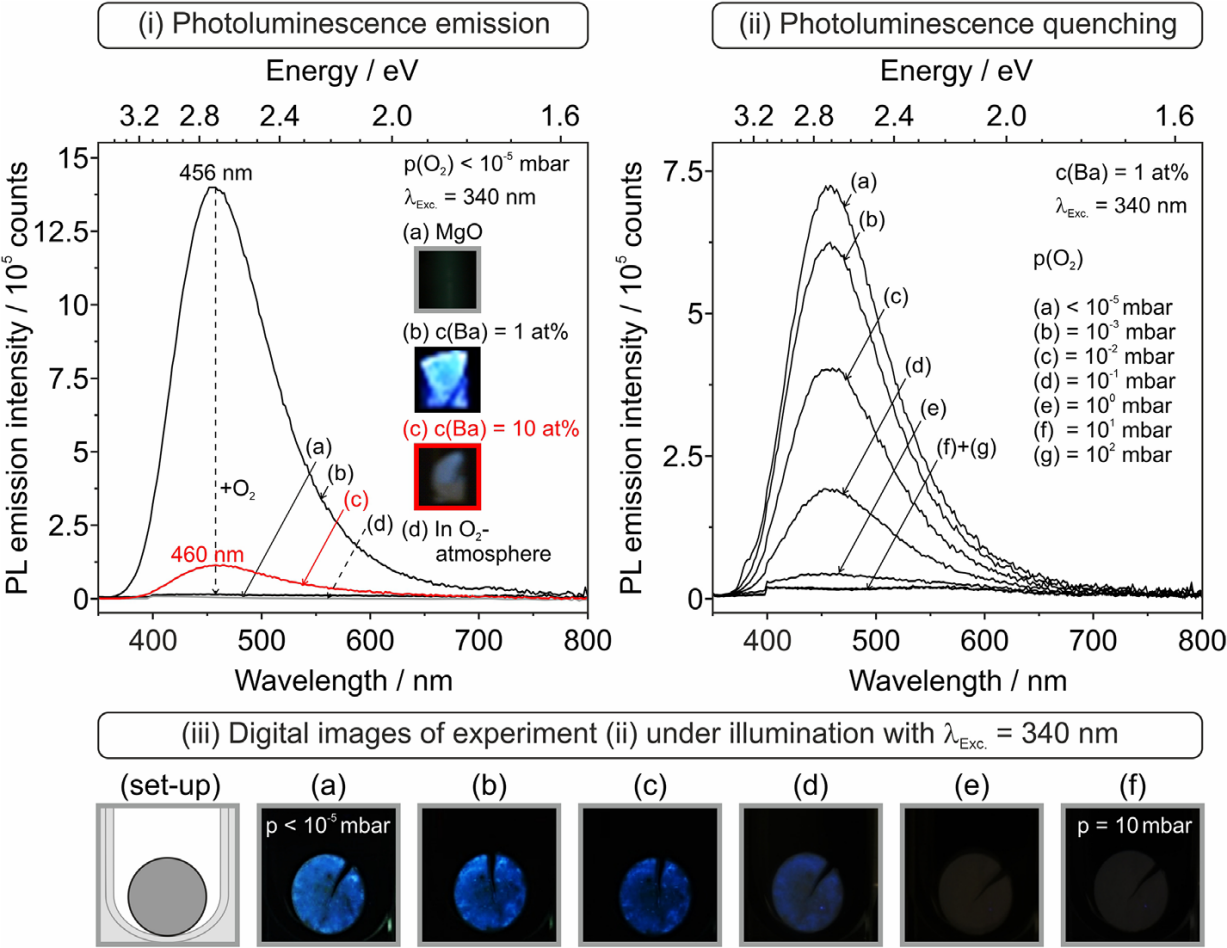
(i) Photoluminescence (PL) emission spectra of sintered (1373 K) and surface dehydroxylated MgO compacts (a) compared to compacts with Ba‐concentrations of 1 at% (b) and 10 at% (c). Spectra (a–c) in the left panel were recorded upon sample excitation at *λ* = 340 nm (3.65 eV) and at *p*(O_2_) < 10^−5^ mbar or (d) in the presence of oxygen gas (*p*(O_2_) = 10 mbar). PL emission from a *c*(Ba) = 1 at% compact was measured in the O_2_ partial pressure range 10^−5^ mbar ≤ *p*(O_2_) ≤ 10^2^ mbar. The digital photographs in (iii, at the bottom) illustrate the O_2_ pressure‐dependent PL emission effect. Appropriate image correction was performed for the elimination of stray light.

With this information, we turn now to the Ba–O compact‐specific PL emission properties after annealing to 1373 K (Figure 9).

As outlined for powders along with the discussion of Figure 7, excitation at *λ* = 340 nm exclusively addresses structural features that contain Ba^2+^ ions, either atomically dispersed inside an MgO surface element (Figure 7F) or as nanocrystalline BaO clusters and islands (Figure 7G). The following key observations can be made:

The absence of the emission feature at *λ* = 500 nm (such as that shown in the right panel of Figure 7 as Figure 7b) suggests that the signal observed exclusively stems from BaO clusters and islands (Figure 7G). Enhanced ion diffusion, grain coarsening, and BaO accumulation in specific regions have led to a microstructure with a negligible abundance of isolated Ba^2+^ surface ions as PL emission sites (Figure 7F).

For compacts with a Ba‐concentration of 1 at%, the PL emission feature centered at *λ* = 460 nm is by orders of magnitude more intense than the one of the 10 at% compacts. From this we conclude that there remains a substantially reduced total number of coarsened BaO exsolutes distributed over the sintered microstructure of the 10 at% Ba compact that hosts BaO edges and corners as PL emission sites (Figure 7G, ^27^, ^34^).

With respect to pressure, O_2_ gas quenches reversibly the BaO‐specific emission features in the range 10^−5^ mbar ≤ *p*(O_2_) ≤ 10^2^ mbar (Figure 9ii and 9iii). This is explained by the collisional deactivation of photoexcited surface states by molecular O_2_ from the gas phase.^35^ Complete quenching of radiative deactivation is achieved at *p* ≥ 1 mbar O_2_ (Figure 9e). Consequently, through the residual porosity of the compacts, all PL emission sites are subject to collisional quenching by O_2_ gas. BaO segregates that become enclosed in between the grains and incorporate into the intergranular films and grain boundaries, however, do not contribute to PL emission anymore.

### 3.6 Ba^2+^ distribution inside the sintered compacts

As the EM analysis reveals, sintering at 1173 K is insufficient to achieve a dense microstructural network consisting of grains, intergranular regions, and grain boundaries inside the Ba*_x_* Mg_1−_ *_x_* O (*c*(Ba) = 10 at%) compacts. At this temperature, dwell times of 2.5 h generate just a few local regions that show particle fusion and exhibit sintering necks (Figure S7). Even extended annealing for 25 h did not produce noticeable changes in the abundance of particle contacts and grain boundaries and in coarsening.

**FIGURE 10:**
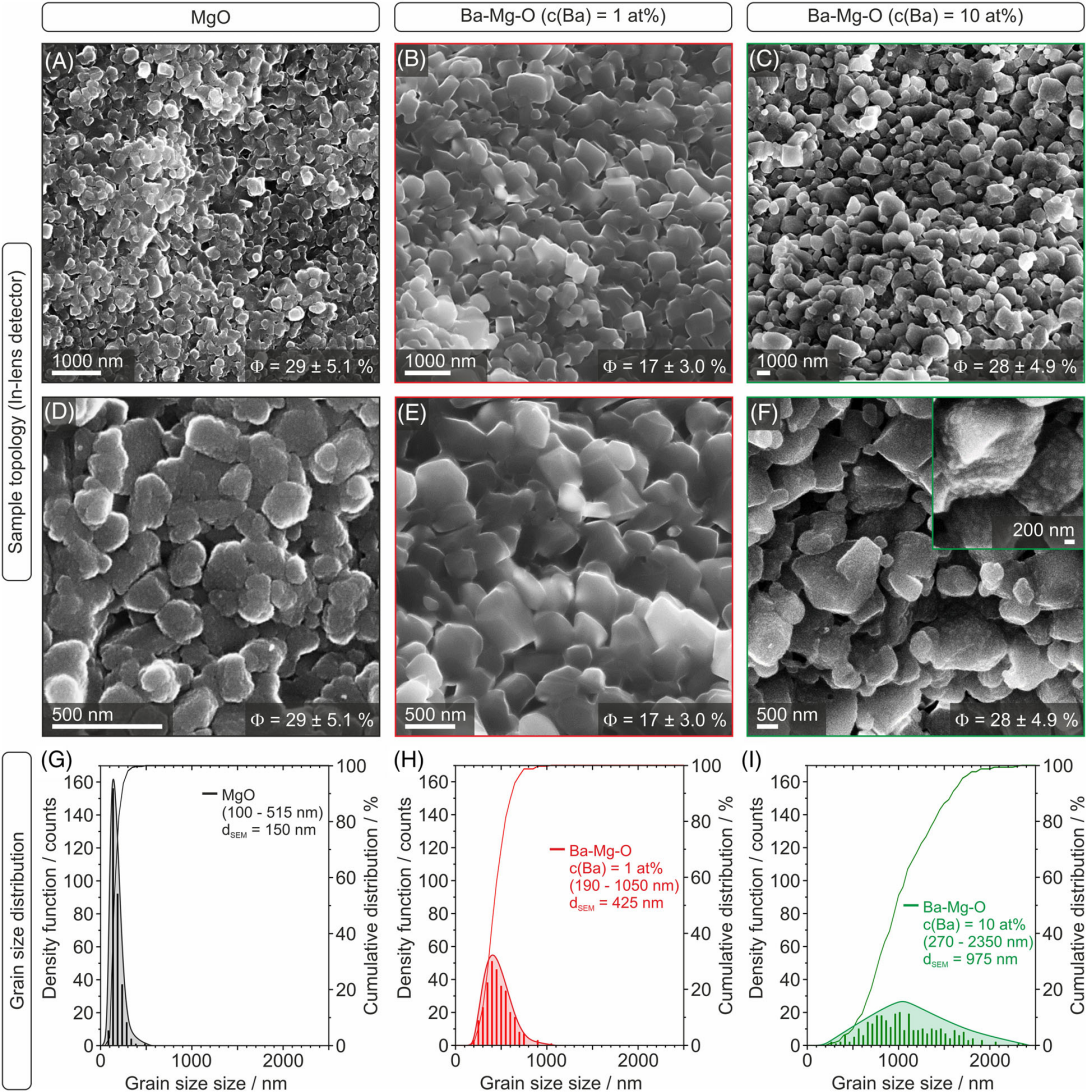
Low‐magnification (first row) and high‐magnification (second row) scanning electron microscopy (SEM) images of fracture surfaces after compact sintering at 1373 K for 2.5 h. Grain topology and corresponding size distribution plots of MgO compacts (A, D, G, left column) are compared to those of Ba*_x_* Mg_1−_ *_x_* O with a nominal Ba‐concentration of 1 at% (B, E, H, middle column) and 10 at% (C, F, I, right column). Values for the residual porosity are provided in the bottom right corner of the images.

Based on this information, we performed the subsequent sintering step at 1373 K. Fracture surface image analyses (Figure 10) of sintered Ba*_x_* Mg_1−_ *_x_* O compacts were compared to those of MgO as a reference. Sample topology (Figure 10A–F, first and second rows) and corresponding grain size distributions (Figure 10G–I, third row) were analyzed as a function of Ba dispersion.

Sintering at 1373 K is associated with substantial grain coarsening and promotes the formation of grain contacts and boundaries inside the emerging microstructure. Low‐magnification SEM images (Figure 10A–C, top row) are consistent with the trend of the residual porosity values (Figure 10, bottom right corner). Interestingly, the densification behavior of MgO (Figure 10A) and Ba*_x_* Mg_1−_ *_x_* O compacts with *c*(Ba) = 10 at% (Figure 10C) with residual porosities around *Φ* = 29% are similar, whereas the 1 at% Ba*_x_* Mg_1−_ *_x_* O compact (Figure 10B) exhibits a denser and at the same time more homogeneous structure. Higher magnification images (Figure 10D–F, second row) reveal trends in grain size and morphology evolution that clearly depend on the concentration and, thus, the dispersion of admixed barium (Figure 10). MgO grains with a relatively narrow size distribution that peaks at 150 nm (Figure 10G) show less‐defined morphologies that deviate from the cubic habit specific to the nanocrystalline source material (Figure 4A). Remarkably, Ba‐admixture at concentrations as low as 1 at% gives rise to a more cubical granular structure with rounded edges (Figure 10E). The size distribution characterized by a mean grain diameter of 425 nm is strongly broadened (Figure 10H).

Moreover, related SEM images reveal finely dispersed surface segregates that are attributed to BaO (Figure 10F including inset). At the present, the complex and multiple influences of Ba segregation on grain reorganization and densification during sintering remain unresolved, and the question why lower amounts of admixed Ba give rise to stronger densification and—at the same time—more characteristic grain habits than higher Ba‐concentrations requires further investigation.

**FIGURE 11:**
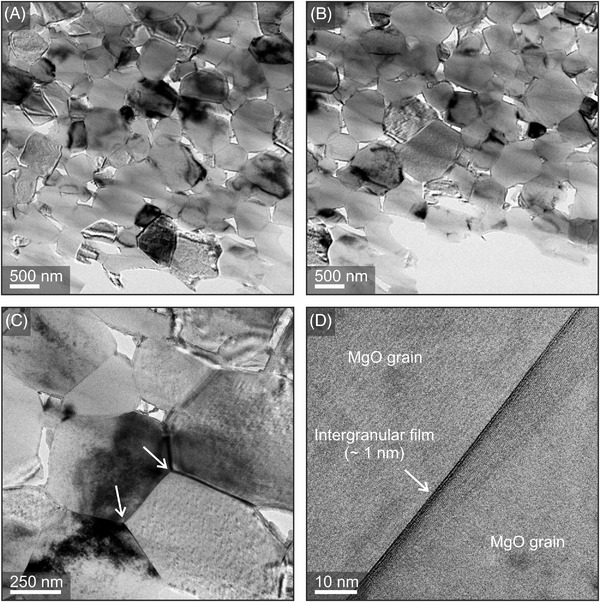
Microstructural analysis of a Ba*_x_* Mg_1−_ *_x_* O compact with *c*(Ba) = 1 at% after sintering at 1373 K and in O_2_/Ar flow. Low‐magnification transmission electron microscopy (TEM) images (top row A and B) reveal the formation of a porous intergranular network. Characteristic microstructural features, including triangular pores, triple junctions, and intergranular films (C) and (D)

The microstructure of a thinned compact with a nominal Ba‐concentration of 1 at% after sintering at 1373 K was accessed with TEM (Figure 11).

**FIGURE 12:**
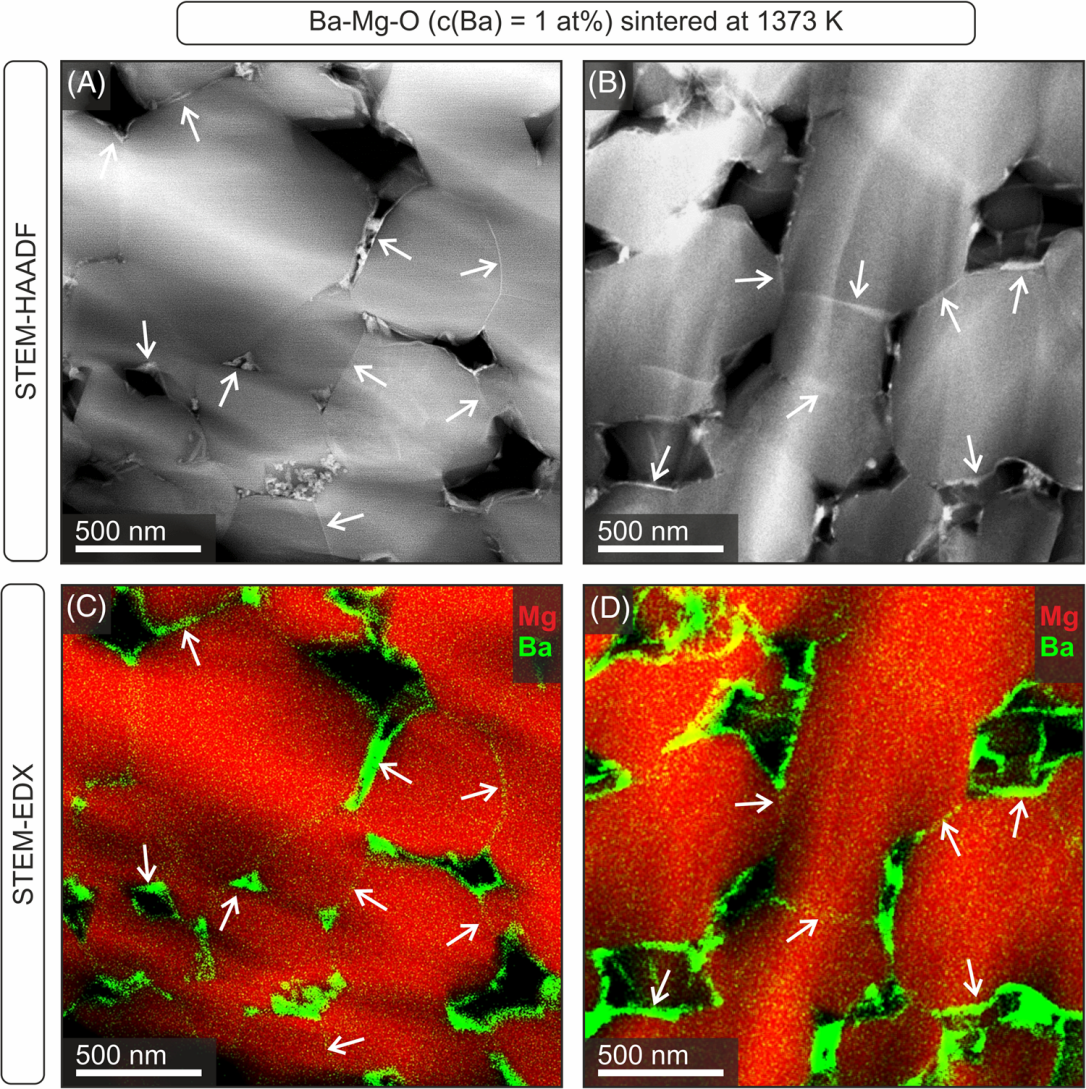
STEM evidence for Ba‐enrichment in the intergranular regions and Ba accumulation at free pore surfaces. Analysis was performed on a Ba*_x_* Mg_1−_ *_x_* O compact with a Ba‐concentration of 1 at% and after sintering at 1373 K. STEM–HAADF images (top row A, B) reveal regions of higher z‐contrast that originate from Ba. These observations are consistent with EDX intensity maps in the bottom row (C, D; red: Mg, green: Ba).

Low‐magnification TEM images (Figure 11A) reveal the porous ceramic microstructure that has evolved. Differences in contrasts depend on the residual thickness of the thinned specimen and the crystallographic orientation of individual grains. Characteristic structural features, such as triangular pores, triple junctions (Figure 11C) grain boundaries, or intergranular films with thicknesses around 1 nm (Figure 11D) can be observed. Compositional analysis addressing the distribution of BaO within the ceramic microstructure was performed in the STEM mode (Figure 12).

In the STEM–HAADF images (Figure 12A,B), regions of enhanced elemental contrast are rich in barium, which is consistent with EDX intensity maps recorded in these regions (Figure 12C,D). Corresponding images clearly reveal Ba segregation and enrichment in the free pore surfaces of the MgO‐based grains. Thereby, Ba exsolution into free pore surfaces where Ba ions form larger BaO clusters seems to be energetically favored as we collected more evidence for this type of segregation product than for Ba‐enriched intergranular films and grain boundary structures (Figures 11D and 12A,B). Finally, we want to emphasize that it is the open pore region that exclusively hosts the PL‐active BaO clusters and that is accessible to the gas phase (Figure 9B,C).

## 4 CONCLUSIONS

We investigated BaO exsolution from Ba*_x_* Mg_1−_ *_x_* O nanoparticle powders and compacts and addressed the influence of the concentration of the segregating species on microstructure evolution, that is, on grain coarsening and densification. Both grain growth and densification exhibit a significant dependence on Ba content, which as an observation could have been decoupled from water and other adsorbate‐induced effects, as sample treatment was performed either in vacuum or in dry Ar/O_2_ atmosphere. Annealing temperature and Ba‐concentration determine the amount of Ba^2+^ ions that either reside isolated inside MgO grain surface elements or accumulate to form nanocrystalline BaO segregates inside the open pore structure of the compact. Compact annealing at 1373 K leads to white and self‐supporting pellets with a residual porosity of about 29%. This enables the interaction between gas molecules and photoluminescent BaO‐specific nanostructures inside the pores that produce bright PL emission in the range of blue light (i.e., with an emission maximum at *λ*  = 460 nm). Within the matrix of MgO‐based grains, Ba‐admixture concentrations of a few atomic %—trapped within nanocrystals in the size range of <10 nm as source material—are effective in generating a sufficient concentration of highly dispersed BaO segregates with an observable PL light emission. Higher BaO‐accumulation inside the pores significantly decreases the dispersion and number of PL active sites.

The approach to utilize Ba^2+^ ion exsolution from the bulk of the Ba*_x_* Mg_1−_ *_x_* O nanoparticles that were produced by chemical vapor synthesis into grain surfaces and interfaces can be extended to host oxides different from MgO. The large ionic radius of Ba^2+^ and, therefore, a size mismatch between Ba^2+^ and the cations of the host lattice drives ion exsolution. The surface‐specific PL activity of nanocrystalline BaO that decorated thermally stable substrates and its high basicity render related segregates to an interesting class of spectroscopic interface probes or active sites for inorganic phosphors, electroceramics, or catalyst components.^18^, ^36^

## AUTHOR CONTRIBUTION

Thomas Schwab (leader) and Oliver Diwald (leader, advisor) designed the research plan. Thomas Schwab, Hasan Razouq (contributor), Korbinian Aicher (contributor), and Gregor A. Zickler (contributor) were responsible for the acquisition and analysis of the data. Thomas Schwab, Hasan Razouq, and Oliver Diwald analyzed and interpreted the data. Thomas Schwab and Oliver Diwald drafted the paper. All authors revised the paper critically and gave their approval to the submitted and final version.

## CONFLICT OF INTEREST

The authors declare no conflict of interest.

## Supporting information

Supporting Information
