# Supplementary material for: Conversion of MgO nanocrystal surfaces into ceramic interfaces: Exsolution of BaO as photoluminescent interface probes
Source: J Am Ceram Soc. 2022 Oct 26;106(2):897–912. doi: 10.1111/jace.18833 (PMC10092509; doi:10.1111/jace.18833)
Supplement: Supplementary file 1 — Supporting Information [file JACE-106-897-s001.docx]

Conversion of MgO Nanocrystal Surfaces

into Ceramic Interfaces: Exsolution of BaO as Photoluminescent Interface Probes

*Thomas Schwab^1,#^, Hasan Razouq^1,#^, Korbinian Aicher^1^,*

*Gregor A. Zickler^1^, and Oliver Diwald^1^**

*^#^*These authors contributed equally to this work

Email: oliver.diwald@plus.ac.at

^1^ Department of Chemistry and Physics of Materials, Paris-Lodron Universität Salzburg, Jakob-Haringerstrasse 2a, 5020 Salzburg, Austria

KEYWORDS; chemical vapour synthesis, nanocrystalline ceramics, segregation, BaO, surface excitons, photoluminescence, cathodoluminescence;

**Supplementary Materials**


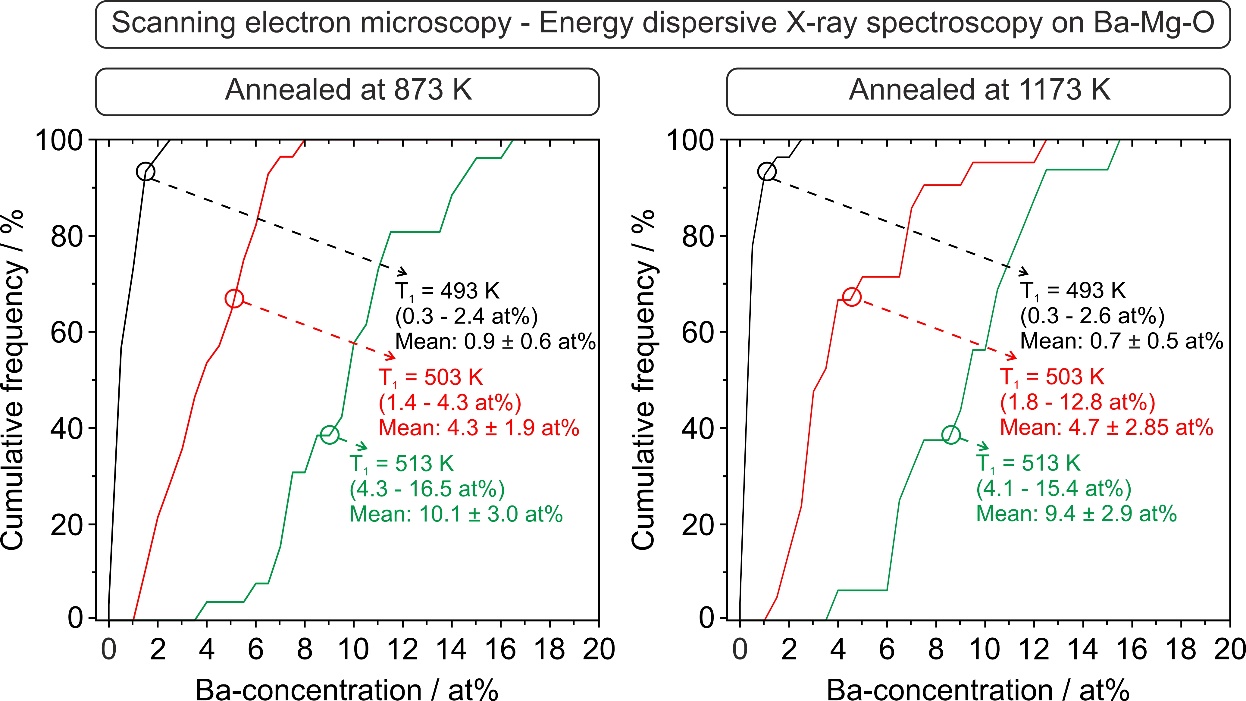


**Figure S1**: Cumulative Ba concentration plots in nanoparticle powders that were produced with different Ba precursor admixtures as controlled by the temperature T_1_ of the Ba precursor reservoir.

| T_1_ / K | Thermal treatment | Ba-concentration / at.% | |
| --- | --- | --- | --- |
| 493 | VA873 | | 1.8 ± 0.6 |
|  | VA1173 | | 1.2 ± 0.2 |
| 503 | VA873 | | 3.5 ± 1.4 |
|  | VA1173 | | 2.9 ± 0.9 |
| 513 | VA873 | | 7.4 ± 1.6 |
|  | VA1173 | | 9.5 ± 1.3 |

**Table S1**: Ba concentration of Ba_x_Mg_1-x_O nanoparticle powder samples that were determined by EDX spectroscopic analysis. The samples were synthesized using different Ba-precursor evaporation temperatures (T1) in the reactor system and, thus, employing different Ba precursor evaporation rates. The powders were vacuum annealed (VA) to 873K (VA873) and 1173K (VA1173). The indicated errors that associated with each value were determined on the basis of the variation of the concentrations that were found in at least three different positions of the powder specimen as analysed with energy-dispersive X-ray (EDX) spectroscopy in conjunction with SEM.


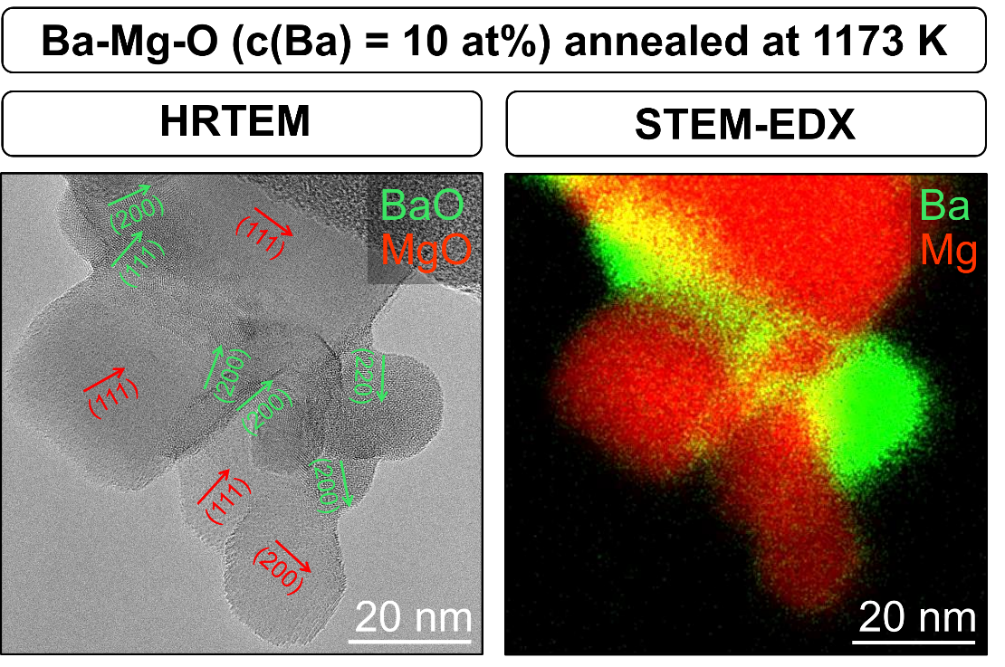


**Figure S2:** High resolution TEM (HRTEM) micrograph (left) and corresponding STEM–EDX intensity map (right) acquired on a vacuum annealed (T=1173 K) Ba_x_Mg_1-x_O powder sample with a Ba-concentration of 10 at%. The HRTEM image shows crystalline regions, where the lattice spacings are consistent with those of crystalline MgO (red) and BaO (green). The surface normal of the lattice planes of the individual single crystalline regions are visualized with red arrows for MgO and green arrows for BaO. The local position of the BaO segregates that were identified on the basis of the characteristic lattice spacings are perfectly in line with the EDX map that reveals specific clusters and regions of Ba exsolutes.


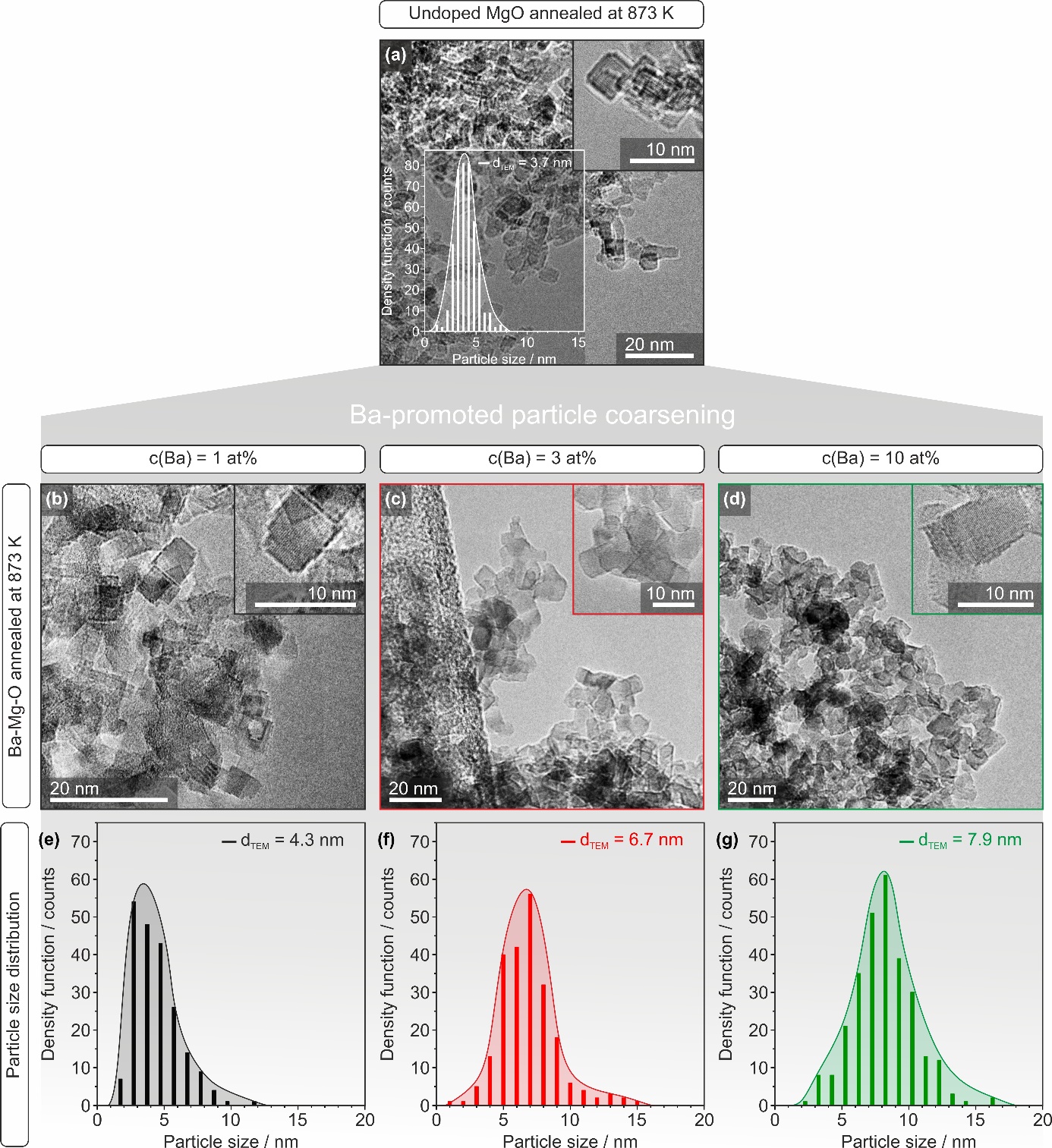


**Figure S3**: Transmission electron micrographs acquired on a pure MgO nanoparticle powder (a, top) and compared to those of Ba_x_Mg_1-x_O nanoparticle powder samples (second and third row, b-g), which contain 1 at% (left column), 3 at% (middle) and 10 at% Ba (right column). The effect of Ba-admixture induced grain coarsening is already observable for powder annealing at T = 873K as demonstrated by the moderate increase in grain size from e, f to g.


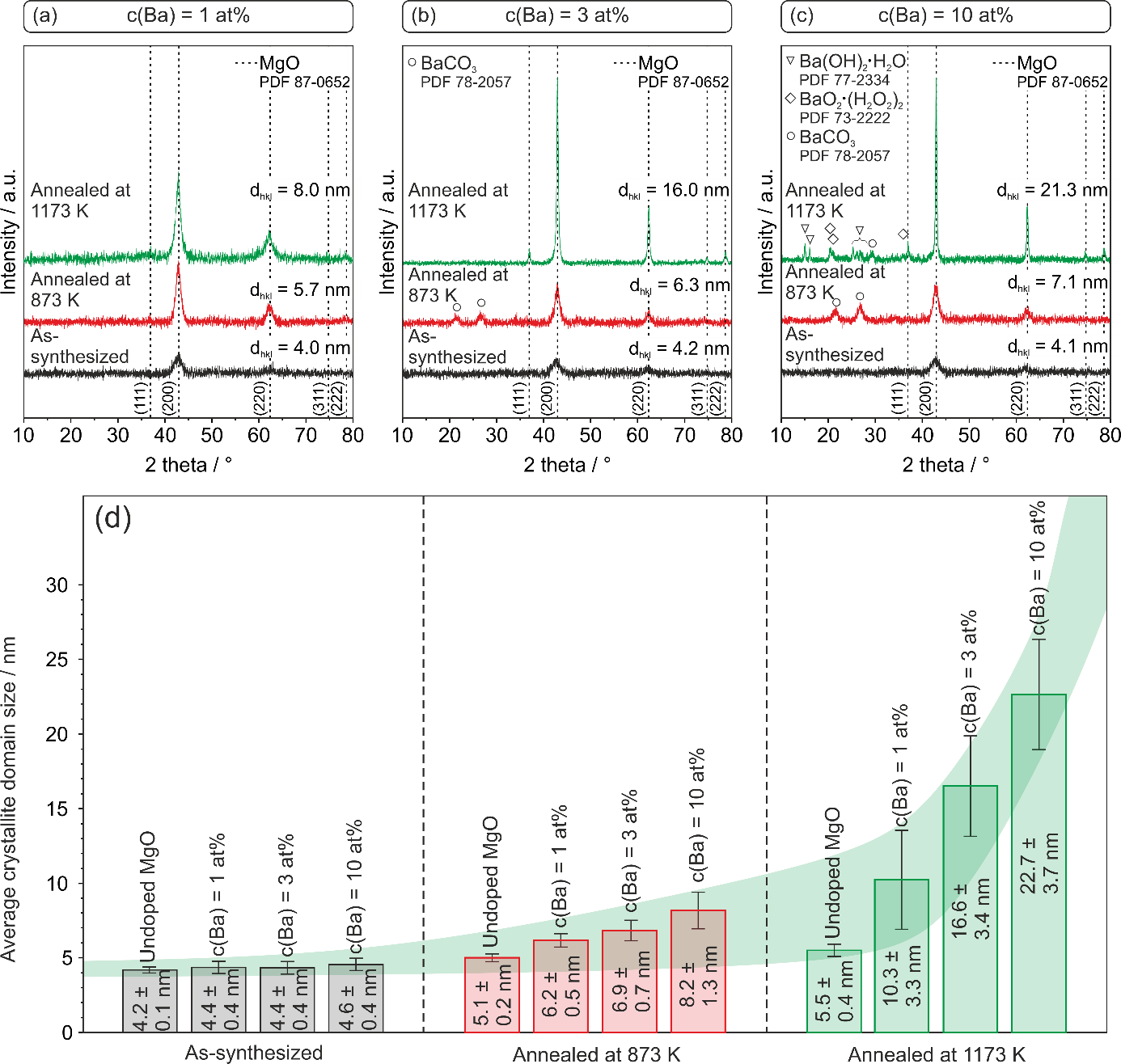


**Figure S4**: Powder X-ray diffraction pattern (top row, a-c) of Ba_x_Mg_1-x_O nanocrystals with Ba-concentrations of 1 at% (left column, a), 3 at% (middle column, b) and 10 at% (right column, c). The diffractograms are organized from bottom to top: as-synthesized (bottom), after powder annealing at 873 K (middle) or 1173 K (top). Corresponding crystallite domain sizes (d) were evaluated by analysis of the (200) related reflection widths using the Scherrer equation. Mean values for the crystallite domain size of all investigated Ba_x_Mg_1-x_O samples are given in (d, bottom) and are compared to those of undoped MgO nanocrystals.

XRD reflection features that appear in addition to those of MgO were observed for Ba_x_Mg_1-x_O samples annealed at 873 K (c(Ba) = 3 at%, c(Ba) = 10 at%) and 1173 K (c(Ba) = 10 at%) (**Figure S4a**) They originate from adsorption of H_2_O, CO_2_, and O_2_ upon sample contact with ambient atmosphere. In the course of XRD measurements this leads to the chemical transformation of surface oxides into hydroxides, peroxides and carbonates. (Schwab et al. 2020b; Ali und Winterer 2010; Winterer und Hahn 2003)

The bottom part of **Figure S4** summarizes the trends of crystallite domain size values with the different Ba-concentrations (1 at%, 3 at%, 10 at%) and annealing temperatures (873 K and 1173 K). Whereas, as-synthesized MgO and the Ba-containing MgO powders exhibit absolutely comparable crystallite sizes in the range below 5 nm, Ba admixture promotes annealing-induced particle coarsening, which - as an effect - increases with annealing temperature. This development is reflected by crystallite domain sizes between 6-8 nm and 10-23 nm for Ba-containing particle ensembles after annealing at 873 K and 1173 K, respectively (**Figure S4d)**.


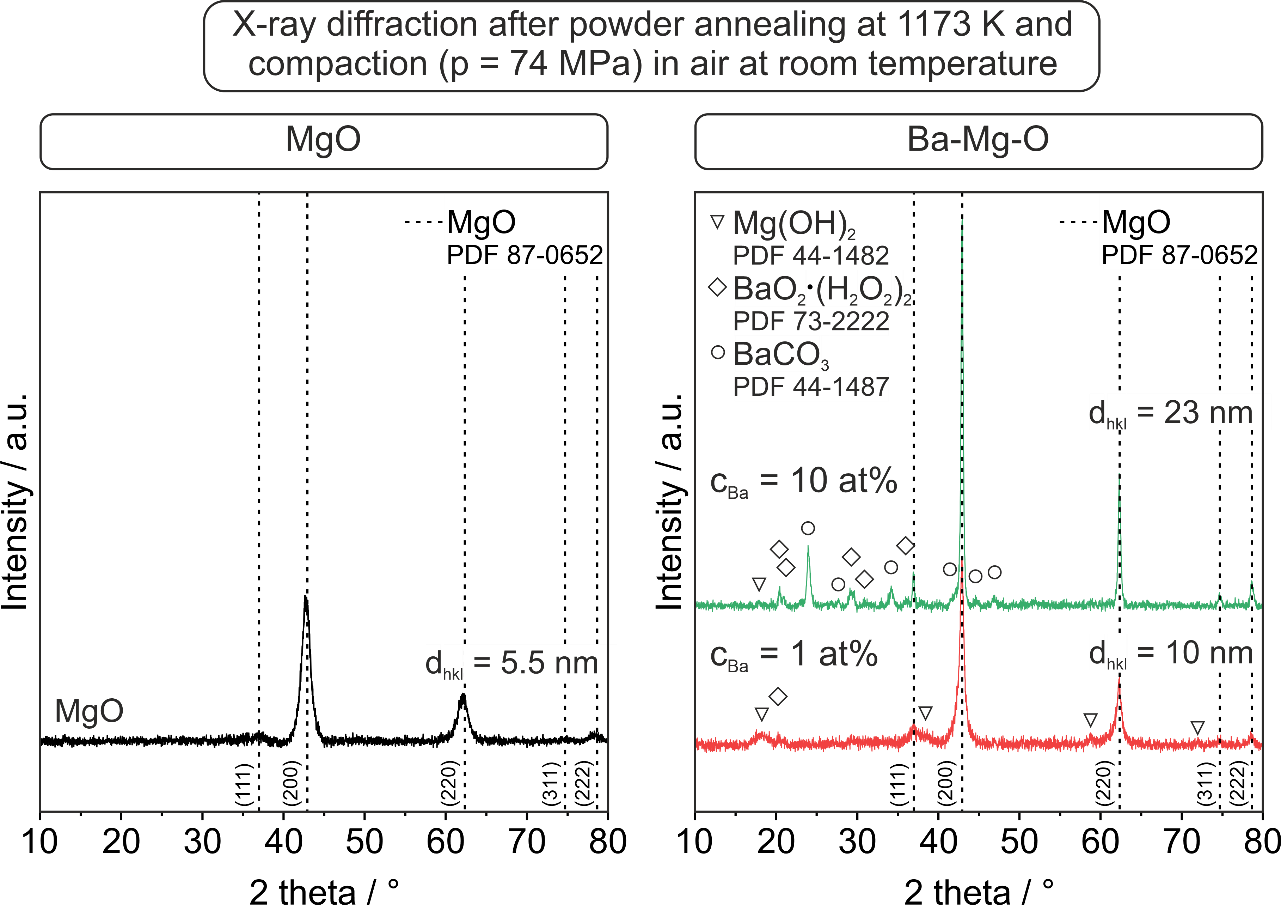


**Figure S5**: X-ray diffraction patterns of MgO (left) and Ba_x_Mg_1-x_O nanoparticle powder compacts (right) with Ba-concentrations of 1 at% and 10 at%. Compaction of pre-annealed powder samples was performed in air and at room temperature. Crystallite domain sizes were obtained by applying the Scherrer equation to the (200) related diffraction feature.

**Figure S5** shows XRD results obtained on pure MgO powder compacts (left) and compares them to those of Ba_x_Mg_1-x_O samples with Ba concentrations of c(Ba) = 1 at% and 10 at% (right). An in-detail discussion of observed compaction induced changes of MgO and Ba_x_Mg_1-x_O nanoparticle powders is provided in reference (Schwab et al. 2020b). Compaction of MgO powders (**Figure S5** left) does not affect the cubic-rock salt specific reflection features and the average crystallite domain size remains as small as 5.5 nm. Within the investigated set of compacted Ba_x_Mg_1-x_O samples (**Figure S5***,* right), a more complex phase information is observed. Together with an apparent increase in the crystallite domain sizes (Schwab et al. 2020b) of starting vacuum-annealed powders from 7 nm and 21 nm (not explicitly shown in **Figure S5)** up to 10 nm and 23 nm for the related compacts, respectively, there are also phase changes that apparently occur during powder compaction in the ambient. These include the formation of additional and hydroxide and/or carbonate related diffraction features that, again, emerge upon the unavoidable contact with water-vapor containing atmosphere during powder compaction and XRD measurements. The conversion into hydroxide or carbonate structures decreases the width in the MgO related diffraction features, an effect that was rationalized in an earlier study by size-dependent dissolution and recrystallization events within nanocrystalline powder materials of enhanced surface basicity.(Schwab et al. 2020b)

To investigate the influence of sintering temperature and atmosphere on both structural and functional properties, the manufactured green compacts were subjected to two different approaches within this study:

1. Annealing and sintering at 1173 K for 2.5 h under dynamic high-vacuum conditions (*p*(O_2_) < 10^-5^ mbar) (**Figure S6a, bottom**)
2. Annealing and sintering at 1373 K inside a muffle-furnace (see detailed information in the experimental section). Here we adjusted constant O_2_/Ar flows to eliminate contributions of residual ambient atmosphere by constantly providing an O_2_ (298 K < T < 1173 K) or Ar-flow (1173 K < T < 1373 K and down to RT) (**Figure S6b, bottom**). After sample annealing in the muffle furnace an additional vacuum-annealing step was applied together with the subsequent admission of oxygen at 1173 K (**Figure S6c, bottom**) to guarantee surface purification and dehydroxylation that is a pre-requirement to perform the reproducible PL-spectroscopy described below (see **Figures S5** and **S7**).

Resulting X-ray diffraction patterns of MgO compacts with Ba admixtures after sintering are compared to those of pure MgO in **Figure S6**.


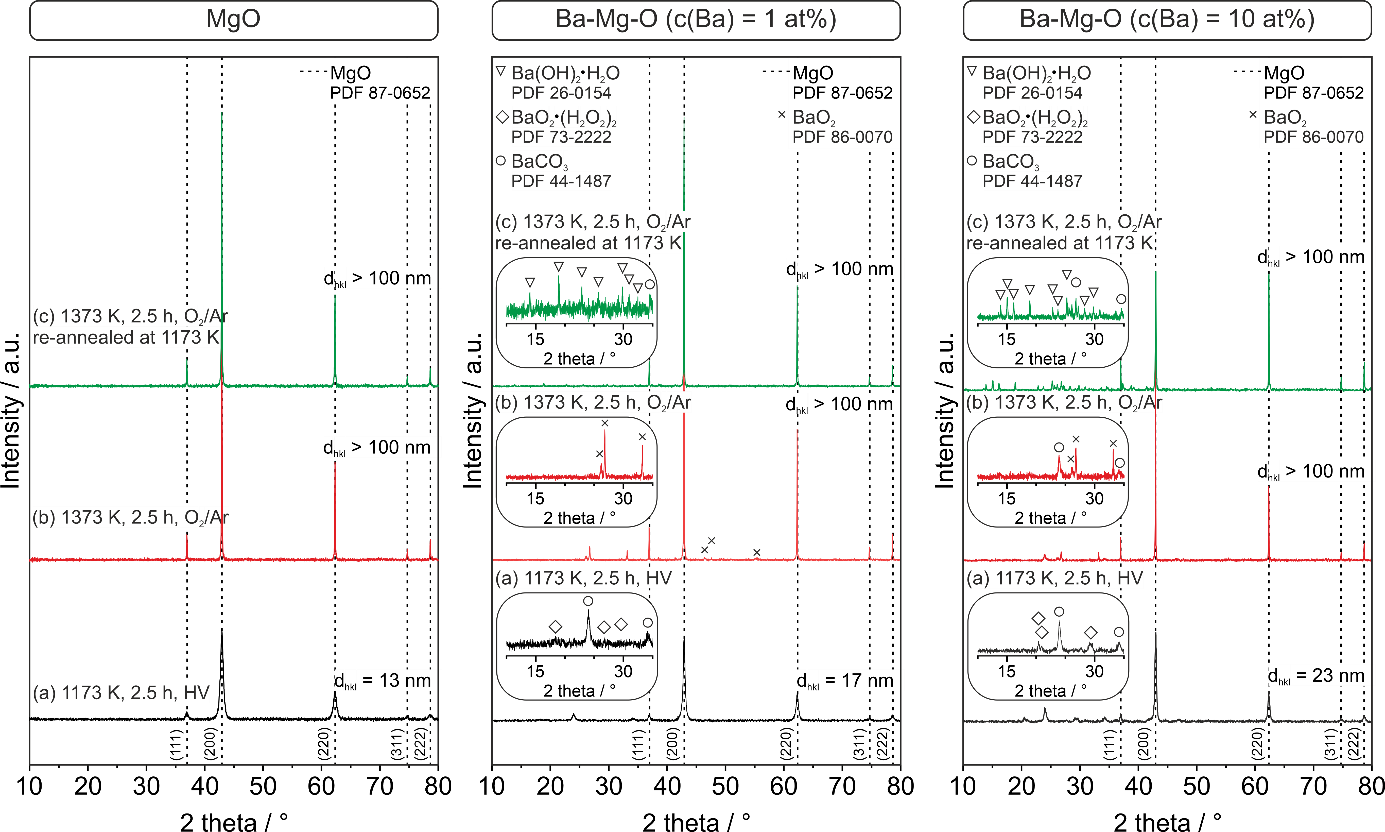


**Figure S6**: X-ray diffraction patterns of sintered MgO compacts (left column) in comparison to sintered Ba_x_Mg_1-x_O compacts (middle and right column) with Ba-concentrations of 1 at% (middle column) and 10 at% (right column). The diffraction patterns are organized from the bottom to the top: (a) bottom: Sintering at 1173 K for 2.5h under dynamic high-vacuum conditions (*p*(O_2_) < 10^-5^ mbar); (b) middle: Sintering at 1373 K for 2.5 h inside a muffle furnace and constant O_2_ or Ar flow; (c) top: re-annealing of (b) under dynamic HV together with sample exposure to oxygen to ensure surface dehydroxylation prior to optical spectroscopy.

Whereas annealing of the green compacts at 1173 K (**Figure S6a** bottom row) promotes moderate coarsening with final crystallite sizes of 13 nm for MgO and 17 nm or 23 nm for Ba_x_Mg_1-x_O compacts with concentrations of *c*(Ba) = 1 at% or 10 at%, respectively, average crystallite domain sizes related to the samples that were subjected to 1373 K inside the muffle-furnace (**Figure S6 b** and **c**, middle and top row) exceed 100 nm and thus the range where the Scherrer equation can be reliably applied. Moreover, high-temperature treatment promotes the emergence of additional reflection features in Ba_x_Mg_1-x_O samples that differ from the MgO specific diffraction lines. They are a direct consequence of the increased surface basicity of the Ba_x_Mg_1-x_O compacts and their interaction with water-containing atmosphere. The diffraction features are attributed to BaO_2_$\cdot$(H_2_O_2_)_2_ and to BaCO_3_ that are stable in vacuum only up to 973 K. In air, these compounds form immediately upon sample contact with O_2_, CO_2_ and H_2_O. Subsequent vacuum-annealing at 1173 K decomposes BaO_2_ specific reflections under formation of barium hydroxide hydrate (Ba(OH)_2_$\cdot$H_2_O) diffraction lines. Structure changes that are subject to temperature changes and/or changes of the gaseous environments during sintering can be clearly observed within this post-mortem XRD analysis performed here. A time-temperature-resolved phase assignment of emerging reflection features, however, requires in-situ studies together with sample measurements in defined gas atmospheres, i.e. an experimental set-up that is not available at the present.

| System | Porosity / % | | | | | | | | |
| --- | --- | --- | --- | --- | --- | --- | --- | --- | --- |
|  | After compaction | | | After sintering for 2.5 h at 1173 K | | | After sintering for 2.5 h at 1373 K | | |
|  | Overall | Closed | Open | Overall | Closed | Open | Overall | Closed | Open |
| MgO | 69 ± 1.8 | 27 ± 3.4 | 42 ± 5.3 | 53 ± 6.7 | 31 ± 6.1 | 22 ± 4.4 | 29 ± 5.1 | 20 ± 4.0 | 9 ± 1.9 |
| c_Ba_ = 1 at% | 69 ± 1.8 | 21 ± 2.6 | 48 ± 6.0 | 39 ± 4.9 | 20 ± 4.1 | 19 ± 3.8 | 17 ± 3.0 | 6 ± 1.3 | 11 ± 2.2 |
| c_Ba_ = 10 at% | 54 ± 1.4 | 23 ± 2.9 | 31 ± 3.9 | 51 ± 6.4 | 21 ± 4.3 | 30 ± 3.9 | 28 ± 4.9 | 13 ± 2.7 | 15 ± 2.9 |

**Tables S2**: Total, closed and open porosity of MgO and Ba_x_Mg_1-x_O ceramics obtained determined for materials at different processing stages and sintering procedures. Porosity values have been calculated from density measurements in combination with He-pycnometry.

**
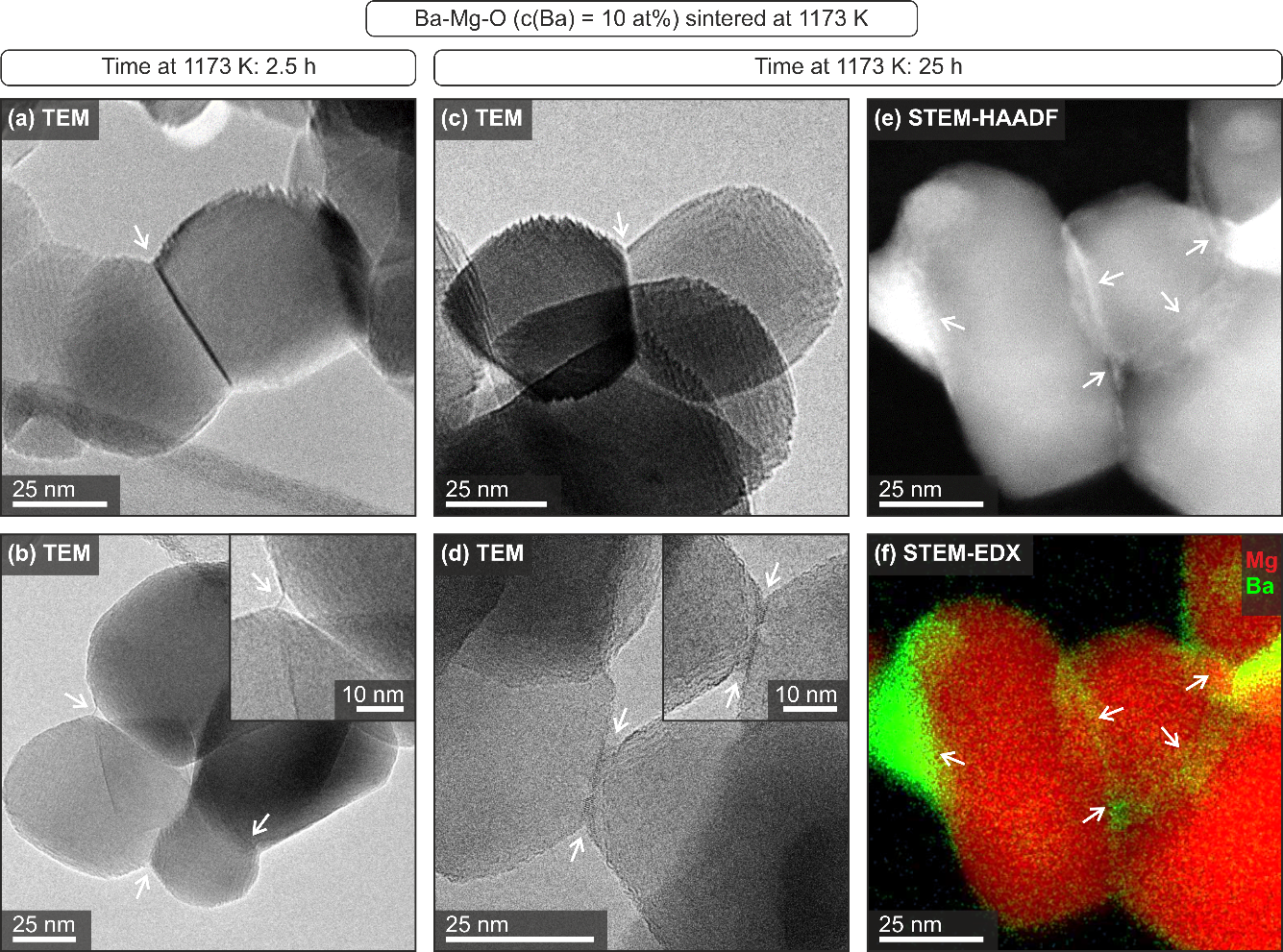
**

**Figure S7:** TEM analysis of a Ba_x_Mg_1-x_O compact with c_Ba_= 10 % at%) after sintering at 1173 K and in dynamic vacuum (p< 10^-5^ mbar). The microstructural investigation was performed on samples after two different annealing times: for 2.5 h (a and b, left and middle column) and for 25 h (c and f, right column). The STEM–HAADF image in e displays compositional contrast that is in good agreement with the STEM–EDX map that reveals Ba-accumulation in intergranular films and surface segregates covering particle surfaces.

With a dwell time of 2.5 h annealing at 1173K (**Figure S7a** and **b** Supplementary Information) generates just a few regions which show particle fusion and sintering necks (inset of **Figure S7b**). Extended annealing for 25 h did not induce substantial changes in the coarsening behavior of the grains and the grain boundary structures (**Figure S7**). It just promotes the ongoing Ba-segregation and the concomitant formation of a Ba rich intergranular regions as accessed by STEM measurements (**Figure S7e** and **f**)

The HAADF-image in **Figure S7e** shows enhanced elemental contrast at both grain surfaces and in the intergranular regions highlighted by white arrows. These regions correspond to Ba-rich segregates as confirmed by the EDX intensity map. BaO structures accumulate in the interfacial region between the grains and as a result of high-temperature treatment.

Elemental contrast images acquired with the angle selective backscatter electron detector (AsB) inside the SEM (**Figure S8**) reveal the higher contrast related to the Ba-rich regions that remain dispersed within the ceramic structure after sintering at 1373 K. These BaO segregates seem to be localized in between the MgO based grains (**Figure S8**, indicated by red arrows). Ba-concentrations as high as 10 at% promote a more inhomogeneous appearance of the entire sample that is composed of small grains of less defined morphology as well as larger and strongly coarsened grains with polyhedral shapes (**Figure S8 c,f**) The latter type of grains also accommodate island-like structures on top (inset of **Figure 8d**). Moreover, the sample is subject to substantial Ba-segregation to form larger Ba-rich regions (**Figure S8**, green circles). Enhanced coarsening results in broadened grain size distributions that reach values as large as 2.3 μm and with a median grain size of 975 nm (**Figure 8**).


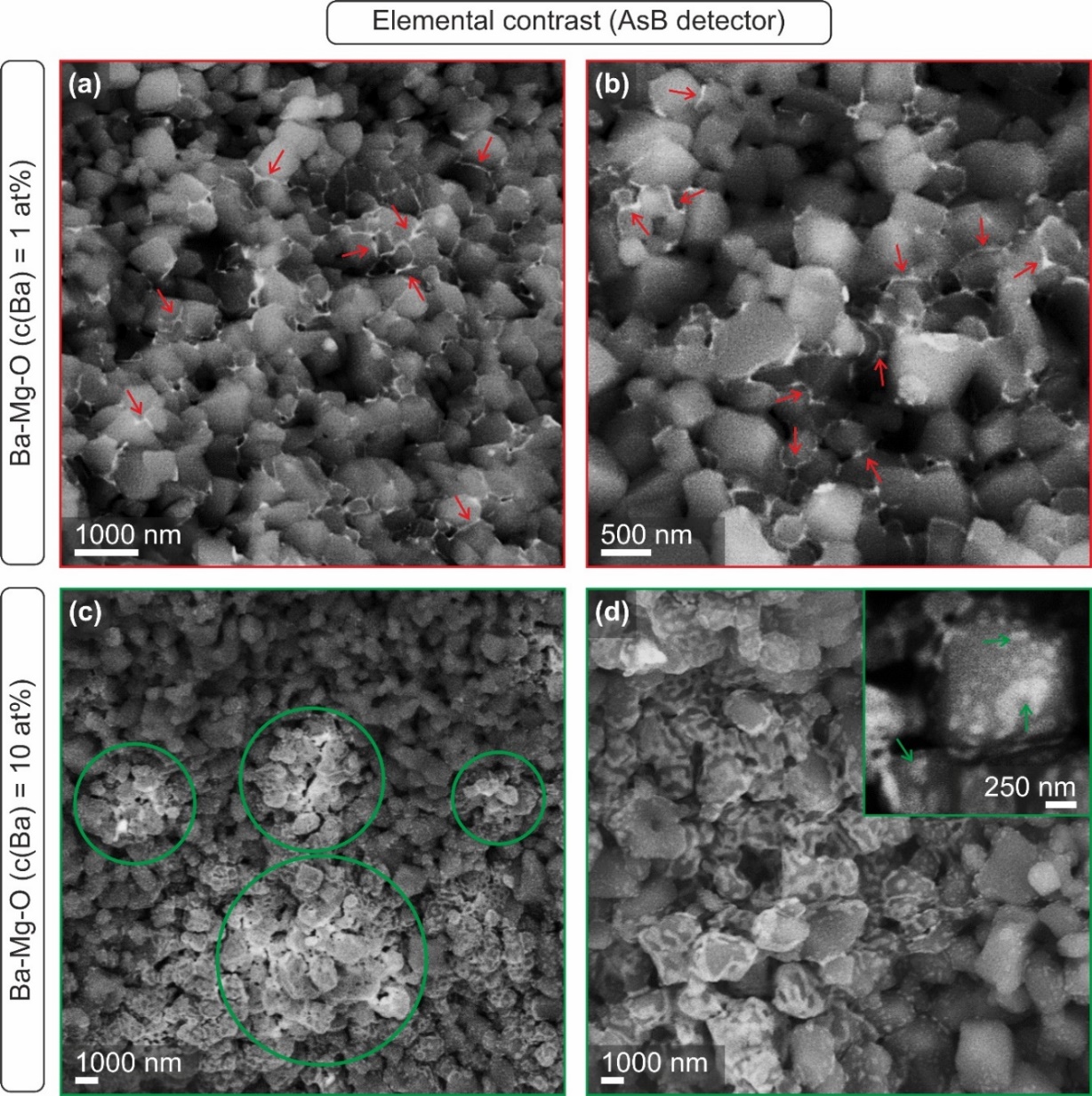


**Figure S8**: Elemental contrast images of ceramic fracture surfaces after sintering at 1373K and with nominal Ba-concentrations of 1 at% (a, b, top row) and 10 at% (c, d, bottom row) acquired with the angle selective backscatter electron (AsB) detector giving z-contrast images inside the SEM. Locations of higher and Ba-related z-contrast appear brighter as indicated by red arrows (top row) and green circles (bottom left). For a low Ba concentration BaO seems to be highly dispersed between the MgO grains whereas larger regions of accumulated BaO clusters that grow on top of the MgO grains (inset in d) are observed for a Ba-concentration of 10 at%.
